# Determination of risk factors for herpesvirus outbreak in oysters using a broad-scale spatial epidemiology framework

**DOI:** 10.1038/s41598-018-29238-4

**Published:** 2018-07-18

**Authors:** Fabrice Pernet, Marine Fuhrmann, Bruno Petton, Joseph Mazurié, Jean-François Bouget, Elodie Fleury, Gaétan Daigle, Pierre Gernez

**Affiliations:** 1 Ifremer, Unité de Physiologie Fonctionnelle des Organisme Marins, LEMAR UMR 6539, Technopole de Brest-Iroise, Plouzané, France; 2 Ifremer, Unité de Physiologie Fonctionnelle des Organisme Marins, LEMAR UMR 6539, Presqu’île du vivier, Argenton, France; 3 Ifremer, Unité Littorale, Laboratoire Environnement Ressource du Morbihan Pays-de-la-Loire, 12 Rue des Résistants, La Trinité-sur-Mer, France; 4 Université Laval, Département de mathématiques et de statistique, Pavillon Alexandre-Vachon, Québec, QC Canada; 5 Mer Molécules Santé (EA 2160), Université de Nantes, Nantes, France

## Abstract

Marine diseases have major impacts on ecosystems and economic consequences for aquaculture and fisheries. Understanding origin, spread and risk factors of disease is crucial for management, but data in the ocean are limited compared to the terrestrial environment. Here we investigated how the marine environment drives the spread of viral disease outbreak affecting The Pacific oyster worldwide by using a spatial epidemiology framework. We collected environmental and oyster health data at 46 sites spread over an area of 300 km^2^ along an inshore-offshore gradient during an epizootic event and conducted risk analysis. We found that disease broke out in the intertidal farming area and spread seaward. Mortalities and virus detection were observed in oysters placed 2 km from the farming areas, but oysters of almost all sites were subclinically infected. Increasing food quantity and quality, growth rate and energy reserves of oyster were associated with a lower risk of mortality offshore whereas increasing turbidity, a proxy of the concentration of suspended particulate matter, and terrestrial inputs, inferred from fatty acid composition of oysters, were associated with a higher risk of mortality. Offshore farming and maintenance of good ecological status of coastal waters are options to limit disease risk in oysters.

## Introduction

Since the mid‐1970s, disease epidemics and mass mortalities have been occurring in marine environments at a historically unprecedented rate^1^ and have economic consequences for fisheries and aquaculture^2^. Understanding where, when and why outbreaks occur and the means by which they spread is crucial for proposing effective disease management. Nevertheless, data on pathogen origin and spread in the ocean are limited^3^. The few cases in which information is available indicate that disease may spread at least as rapidly as any terrestrial epidemic, reflecting, among other things, the relative openness of marine systems compared to terrestrial ones^4^. The most emblematic case is the herpesvirus that spread through pilchard populations in Australia at a rate in excess of 10 000 km year^−1^ ^5^.

The risk of disease outbreak depends on interactions between hosts, pathogens, and the environment, and any change in one or more of these components may potentially increase or decrease this risk^6^. Climate change related factors such as increasing temperature, rainfall anomalies and storms and acidification drive host-pathogen interactions in the marine environment and infectious disease outbreaks affecting corals, shellfish, finfish and humans^6^. Anthropogenic factors like enhanced terrestrial inputs from runoff, nutrient load and pollutants, and aquaculture practices have also significant implications for the emergence and spread of diseases. Ultimately, disease outbreaks in a changing ocean can be shaped by multiple factors acting on the host and the pathogen simultaneously.

Ostreid herpesvirus type 1 (OsHV-1) infecting the Pacific oyster *Crassostrea gigas* has gained considerable attention during the last 10 years due to economic costs associated with increased mortalities in farmed animals worldwide^7,8^. In addition to being socio-economically devastating, oyster diseases affect overall ecosystem productivity and health. Oysters can act as keystone species by providing ecosystem services such as, controlling phytoplankton bloom and turbidity, reducing bank erosion and providing shelters and habitats for other species^9^.

A great deal of work has been done on OsHV-1 risk factors but information gaps still remain^7,8^. One risk factor that is virtually unknown is the potential for long-distance dispersal of OsHV-1 disease. Yet, this is a fundamental prerequisite for building epidemiological models and evaluating the effectiveness of disease management scenarios. In the marine environment, distance over which viruses can be transmitted is generally limited by the interaction of hydrodynamics with viral shedding and decay rates^10^. Decay rates of marine viruses and persistence outside the host vary with environmental factors as seawater temperature, salinity, solar radiation, natural bacterial communities, suspended particulates and grazing^11,12^.

Knowledge gaps remain in the study of persistence of viruses in the marine environment in part as most experiments have been conducted at local scale whereas disease spread and risk factors in the marine environment are affected by processes that occur at broader regional scale such as hydrodynamic circulation and connectivity patterns^4^. Our objective is to investigate the origin and spread of a marine viral disease and to identify risk factors that affect disease dynamics at the regional scale using a spatial epidemiology framework.

Spatial epidemiology consists in the description and analysis of geographical variations in disease to identify risk factors that could explain these patterns^13,14^. Since the first disease maps produced during the nineteen century to identify causes of plague, yellow fever and cholera^15^, spatial epidemiology grew in complexity and utility, and recently benefited from advances in data availability, analytical methods, and popularization of geographic information systems (GIS)^13^. Spatial epidemiology is now playing an increasingly important role in our understanding of the relationship between diseases and the terrestrial environment, but the application of such analytical framework in the marine environment is still in its infancy.

Pacific oysters are well suited for spatial epidemiology because they are sessile, widely distributed in temperate waters and they survive considerable environmental fluctuations^9^. Also, the recent development of specific pathogen free (SPF, low Vibrio load, absence of detectable OsHV-1) oysters has enabled assessment of the infection process under natural conditions^16–18^. The SPF oysters are descendants of a pool of genitors produced under controlled conditions to minimize the influence of genetic and environmental parameters that could affect the host sensitivity to the disease.

In the present study, mortality and OsHV-1 DNA were monitored in SPF *C. gigas* deployed at 46 sites spread over a 300 km^2^ surface area along an inshore-offshore gradient. Seawater temperature, salinity, chlorophyll-*a* fluorescence, turbidity, dissolved oxygen, bacteria and vibrio concentrations and host parameters (bacteria and vibrio concentrations, energy reserves, food quality and growth rate) were regularly measured to investigate disease risk factors. Altogether, this dataset was used to quantify the influence of environmental variability on disease origin and spread in the coastal ocean.

## Method

### Experimental design

SPF Pacific oysters were produced under controlled conditions until they reached the age of 8 months and wet weight of 0.51 g^16–18^ (File S1). The oysters were screened using an OsHV-1-specific quantitative PCR assay^19^ at the different stages of production (3, 7 and 8 months). No OsHV-1 DNA was detected.

**Figure 1:**
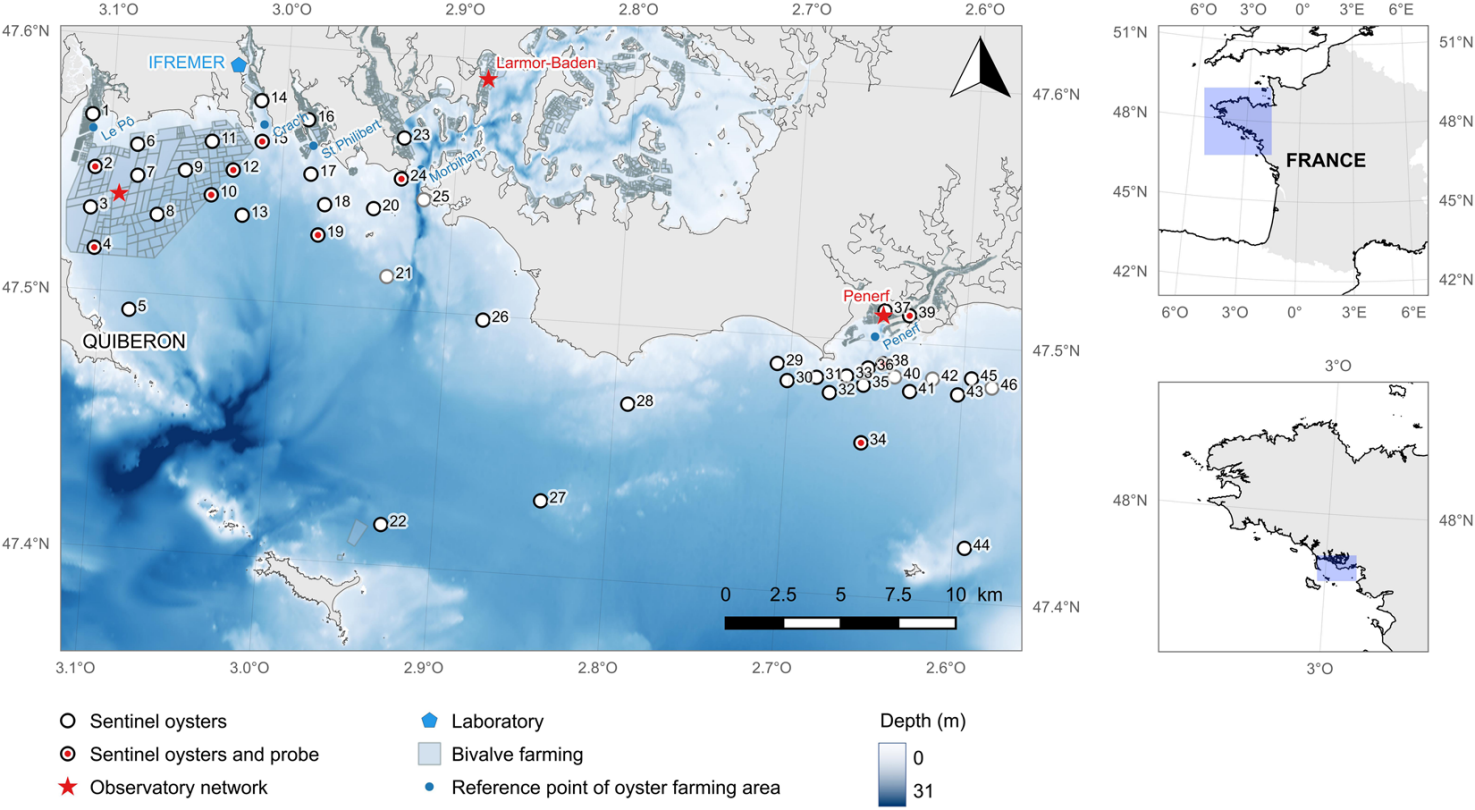
Site map. Sentinel oysters were deployed at 46 sites represented by circles in the Mor-Braz area in southern Brittany, France. The grey circles represent the sites where the sentinel oysters were accidentally lost and where only the seawater data are available. Seawater temperature was measured every 30 minutes during the entire experiment in ten oyster bags evenly spread throughout the study area. In addition, oysters from the shellfish observatory network were monitored at two sites located in the intertidal zone (Pénerf and Larmor-Baden) and at one site in the subtidal zone (Men-er-Roué). This map was done with the open source QGIS ver. 2.18.9. (QGIS Development Team, 2016, https://qgis.org).

The SPF oysters (also called “sentinel oysters”) were deployed at 46 sites located along an inshore-offshore gradient in the Mor-Braz area, South Brittany (France) before the start of the OsHV-1 disease induced mortality event on 8 April 2013 for 171 d until 26 September 2013 (Fig. 1). Five sites were in intertidal oyster farming areas (1, 14, 16, 23 and 37) and 41 sites were offshore. Offshore sites were generally free of oyster farming except in the Quiberon bay where oysters are farmed on-bottom. At each site, 16 small mesh bags containing 85 individual oysters were grouped in one big mesh bag. These bags were attached to iron tables for the sites situated in the intertidal farming area or immersed vertically at 2 meters deep and attached to a mooring point for the sites in the offshore area (File S1). Seawater temperature was measured every 30 minutes during the entire experiment using SP2T recorders (NKE instrumentation, Hennebont, France) placed in ten oyster bags evenly spread throughout the study area (Fig. 1).

Sites were visited 15 times on 30 April, 15 and 27 May, 7, 14, 20 and 27 June, 4, 11, 19 and 25 July, 1, 13 and 31 August, and 26 September by means of two vessels. One boat started from the Ifremer laboratory and covered the Quiberon Bay while the other one started from the Pénerf River (site 39) up until reaching sites 27, 44 and 46 (Fig. 1). Sampling cruises were conducted at low tide slack water ±2 hours and lasted for 3 to 4 hours each.

At each visit, environmental parameters (temperature, salinity, fluorescence, turbidity and oxygen) were measured at the vicinity of the oyster bags using a multiparameter probe (MP6, NKE instrumentation). Data were acquired every 10 seconds over a period of 3–5 minutes per site. During this time, a 50-mL sterile Falcon tube (Dutsher, Issy-les-Moulineaux, France) attached to the frame of the probe was filling with seawater by means of a perforated cap for further bacterial analyses. The seawater sample was screw-capped and stored in a cooler. In a separate cooler, one small bag of oysters was placed and held at ambient seawater temperature.

Upon arrival in the laboratory at la Trinité-sur-Mer, seawater samples were plated on marine broth medium to quantify cultivable bacteria and on thiosulfate-citrate-bile salts-sucrose agar (TCBS) to quantify vibrios (File S2). Living and dead oysters were counted to evaluate survival. Living oysters were divided in three sample groups dedicated to (1) biometry, biochemical analyses and OsHV-1 DNA detection, (2) bacterial analyses and (3) monitoring of survival and detection of OsHV-1 DNA in laboratory conditions at 21 °C.

Individual shell length and wet mass were measured on a sub-sample of 25 living oysters per bag. The soft tissues of these oysters were removed from the shells, pooled together, dipped into liquid nitrogen and stored at −80 °C until laboratory analyses. The remaining shells were dried overnight at room temperature and weighted. Biochemical analyses were conducted on samples collected prior to the mortality event (27 May, 7, 14 and 20 June) whereas OsHV-1 DNA detection were conducted on samples collected prior to and during the mortality event (27 May, 20 June and 13 August). Cultivable bacteria and vibrios were quantified on a sub-sample of 10 alive oysters per bag for every sampling times^17^. Soft tissues were pooled, weighed, homogenized in sterile artificial seawater and plated on marine broth medium for cultivable bacteria and on TCBS for vibrios (File S2).

The remaining oysters (mean n = 42 individuals ±8) from each site were brought back to the Ifremer hatchery in Argenton where they were maintained at 21 °C for 12 d days to reveal subclinical infection i.e. asymptomatic carriers of OsHV-1^17^ (File S3). Oysters collected on 27 May, 7, 14, 20 and 27 June, and 4, 11 and 19 July were tested. These dates corresponded (1) to times of the year when seawater temperature was lower than 16 °C (27 May and 7 June), a threshold temperature above which disease transmission was optimal and mortalities occurred, (2) to the onset of the mass mortality (14 and 20 June) and (3) to the spread of the mortality event (from 27 June onwards). The oysters were placed in 5 L jars (one for each site) filled with UV sterilized and 1 μm-filtered seawater and covered with aluminium foil. All the jars were placed in the same room with controlled air temperature. Seawater was renewed twice a day and a phytoplankton mixture was added at each water renewal (File S3). Dead animals were counted every 0.5 to 2 days. There was no cross-infection among jars (sites), and the mortality events observed under laboratory conditions were associated with OsHV-1 DNA detection (File S3).

The mooring deployed at sites 21, 25 and 42 were accidentally lost at the beginning of the experiment on 15 May. Also, the oyster bags deployed at sites 46 and 40 were lost on 7 June and 14 June respectively. Since the mooring system was still in place at site 46, new SPF oysters were added on 14 June. The data collected at these five sites were not taken into account in the risk analyses. However, the environmental and microbiological parameters of the seawater for these sites were recorded during the entire period of study.

To supplement our experimental design regarding OsHV-1 detection, the temporal evolution of OsHV-1 DNA level in three pools of three oysters collected twice a month from early May to mid-September at two sites in the intertidal zone and at one site in the subtidal in the Mor-Braz area (Fig. 1) obtained by the oyster observatory network is presented here (File S4).

### Laboratory analyses

Pooled oysters (n = 25 individuals) were ground with a MM400 homogeniser (Retsch, Eragny, France) under liquid nitrogen, and the resulting powder was sub-sampled for pathogen detection and biochemical analyses (File S2). The detection and quantification of OsHV-1 DNA was carried out using a real-time PCR protocol^19^. Biochemical analyses consist in the quantification of energetic reserves (carbohydrates and neutral lipids) and fatty acid trophic markers. Carbohydrates were quantified by spectrophotometry and expressed in mg g^−1^ dry mass of tissues. Neutral lipid classes were analysed by high performance thin layer chromatography (File S4). Identified compounds were triacylglycerol (TAG) and sterols (ST). The TAG-ST ratio is a proxy for the relative contribution of lipid reserve to structure. Finally, fatty acid composition of neutral lipids was analysed by gas-chromatography. The selected fatty acid trophic markers are extensively used in ecology^20^ and were related to diatoms (16:1n-7/16:0 and 20:5n-3/22:6n-3); terrestrial inputs (18:2n-6 + 18:3n-3), animal tissues (carnivory, 18:1n-9/18-1n-7); freshness (polyunsaturated/saturated fatty acid, PUFA/SFA), and bacteria (sum of branched chain fatty acids and unbranched 15:0 and 17:0).

### Data analyses and statistics

Analyses were conducted using SAS 9.4 (SAS institute, Carry, USA). The proportion of surviving oysters was analyzed as a function of time for each site. Survival (*S*) was fitted a nonlinear regression model according to the following equations:

1 $$S=\{\begin{array}{c}\alpha \,{\rm{if}}\,d < {D}_{0}\\ \alpha +\beta (d-{D}_{0})\,{\rm{if}}\,{D}_{0}\le d\le {D}_{0}+\delta \\ \alpha +\beta \delta \,{\rm{if}}\,d > {D}_{0}+\delta \end{array}$$

where *α* is the mean survival before the appearance of a mortality event, *d* is the number of days since the deployment of oyster on the field, *D*_0_ is the number of days before the mortality event (also referred to as mortality-free time), β is the daily variation of survival of oysters during the mortality event, and δ is the duration of the mortality event (File S5).

Shell length, total body mass, shell mass and flesh mass of oysters were fitted using segmented regression models according to the following equations:

2 $$Y=\{\begin{array}{c}{b}_{0}+{b}_{1}\frac{d}{7}\,{\rm{if}}\,d < \widehat{{D}_{0}}\,\\ {b}_{0}+{b}_{1}\frac{\widehat{{D}_{0}}}{7}+{b}_{2}\frac{d-\widehat{{D}_{0}}}{7}\,{\rm{if}}\,d\ge \widehat{{D}_{0}}\,\end{array}$$

where *b*_0_ is the intercept, *b*_1_ and *b*_2_ are the regression coefficients before and after the mortality event respectively. The regression coefficients *b*_1_ and *b*_2_ were different for all parameters excepted for total body mass where a simple regression model was fitted to the data (File S6). When no mortality occurred, $\widehat{{D}_{0}}$ was set at the mean value (84.3 d, File S5). Each biometrical parameter was expressed on a weekly basis.

The relationship between the mortality-free time (*D*_0_) and the covariates of interest were investigated using the univariate Cox proportional hazards regression model^21^. These covariates consist of the environmental, microbiological, biochemical and biometrical parameters previously described. When a variable was acquired more than once between deployment and *D*_0_, the mean value and the value recorded before the onset of mortality were used, except for the biometrical parameters where the overall growth rates (the regression coefficient *b*_1_) were used. For the sites where no significant mortality was observed, *D*_0_ was censored and set at 171 d, and only covariate measures taken before 84.3 d were considered (File S5). The relationship between β, δ and the covariates were investigated using Spearman correlation coefficients.

All data generated or analyzed during this study are included in this published article (and its Supplementary Information files).

## Results

### Origin and spread of disease-induced mortality of oysters

**Figure 2:**
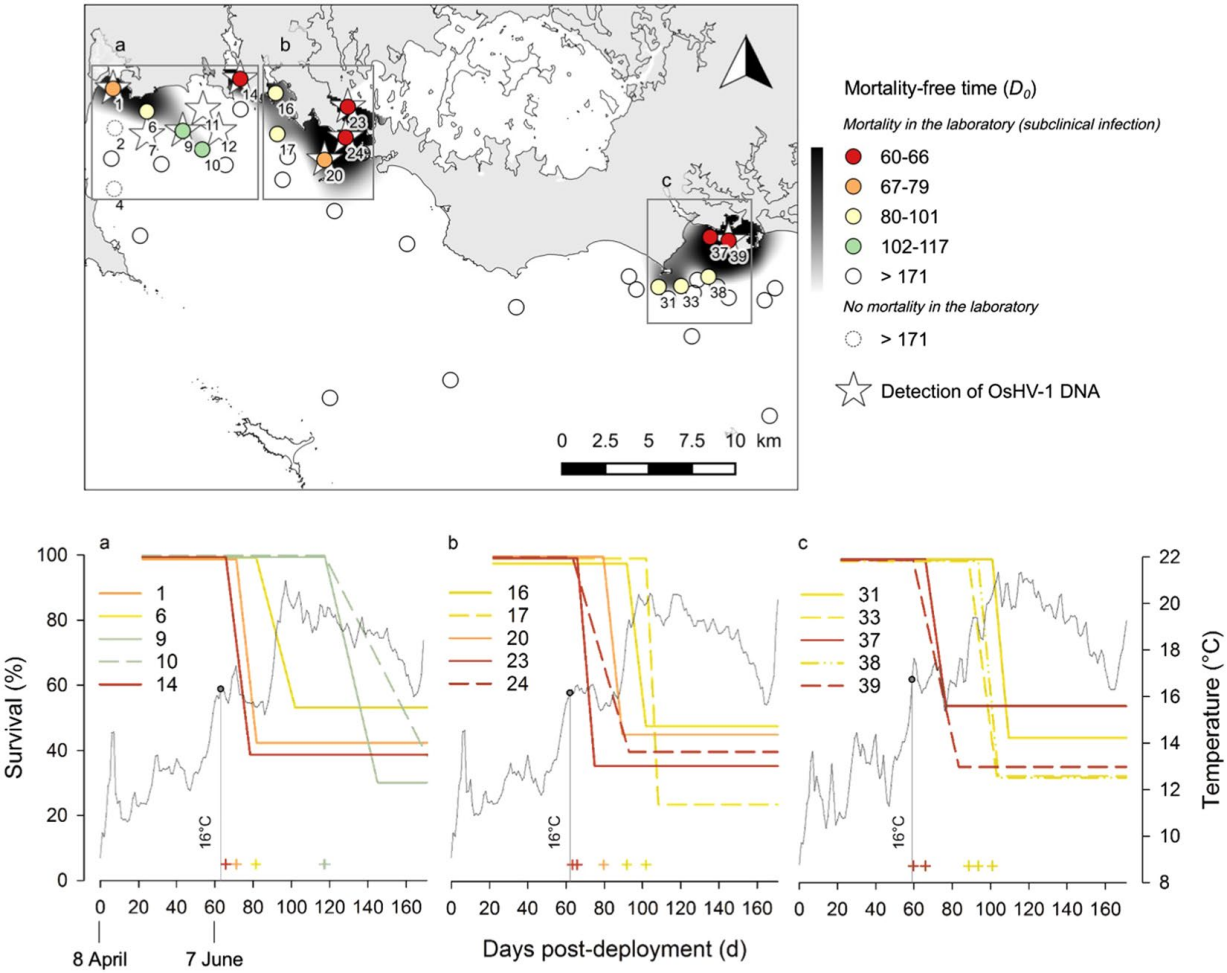
Survival of sentinel oysters in the field, detection of OsHV-1 DNA and subclinical infection. Top: map of the number of days before the mortality event in the field (*D*_0_). For the sites where no mortality was observed, *D*_0_ was reported as greater than 171 d. Plain circles indicate that mortality occurred at least once in the laboratory (subclinical infection) whereas dashed circles show the two sites where no mortality occurred in the laboratory. Bottom: fitted survival curves of the sentinel oysters at the sites where a mortality event was observed (left axis). The plus signs indicate *D*_0_ values for each survival curve. Daily mean seawater temperature recorded in each zone (right axis).

The sentinel oysters were affected by the mass mortality event at 15 sites out of 46 (Fig. 2, File S5). Mortality initiated inshore in oyster farming areas when average daily seawater temperature reached 16 °C (after 7 June, 60 d after deployment), and gradually spread seaward. The number of days before the mortality event (*D*_0_) varied from 60–66 d in inshore oyster farms (sites 14, 23 and 37) and in two adjacent sites (24 and 39), to 117 d offshore (sites 9 and 10) while seawater temperature was higher than 19 °C. The final survival of oysters at these 15 sites varied from 23.7 to 54.2% (mean value of 39.6 ± 8.5% s.d.).

Overall, the spatio-temporal distribution of oyster mass mortality coincided with the general spread of OsHV-1 in oysters. OsHV-1 DNA was not detected in oyster tissues before the mortality outbreak on 27 May (49 d after deployment, averaged seawater temperature 12.8 °C), whereas it was detected on 20 June (73 d, averaged seawater temperature 16.5 °C) in 6 sites where mortalities were either already occurring (sites 1, 14, 23, 24 and 39) or upcoming (site 20, Table 1). Later on, a high level of OsHV-1 DNA (>10^7^ cp mg^−1^) was detected on 13 August (127 d) in oysters from site 9 where mortality had occurred 10 days before the sampling. At the same date, low levels of OsHV-1 DNA (>10^4^ cp mg^−1^) were observed in oyster samples of neighboring sites where no mortality was noticed (sites 7, 11 and 12). OsHV-1 DNA was not detected in sites 6, 10, 16, 17, 31, 33 and 38 where mortality occurred between 82–117 d, suggesting that the detection of OsHV-1 DNA was missed because of the large sampling interval. The data from the oyster observatory network confirms that the mortality (*i*) was associated with the presence of OsHV-1, (*ii*) started in oyster farms located inshore, and (*iii*) spread offshore (File S4).

**Table 1:** Detection of OsHV-1 DNA according to site and time in oysters deployed in the Mor-Braz.

Site	OsHV-1 DNA (cp mg^−1^)		
	27 May (49d)	20 June (73d)	13 August (127d)
1	nd	2.4 × 10^5^	nd
7	nd	nd	1.4 × 10^3^
9	nd	nd	1.3 × 10^7^
11	nd	nd	2.3 × 10^3^
12	nd	nd	7.0 × 10^3^
14	nd	1.8 × 10^5^	nd
20	nd	8.7 × 10^4^	nd
23	nd	7.6 × 10^3^	nd
24	nd	2.3 × 10^5^	nd
39	nd	2.6 × 10^4^	nd
All other sites (n = 36)	nd	nd	nd

Quantification of OsHV-1 DNA was carried out in oyster tissues sampled before, during and at the end of the mortality event on 27 May, 20 June and 13 August respectively. Abbreviation: nd, not detected (<10^2^ cp mg^−1^).

The relationship between oyster mortality and OsHV-1 was strengthened by the results of the challenges performed in laboratory conditions to reveal subclinical infection. When exposed during 12 days to a seawater temperature of 21 °C in the laboratory, the sentinel oysters collected on 27 May (49 d) showed no mortality (File S3). This is consistent with the lack of mortality and of OsHV-1 DNA detection in the field (Fig. 2, Table 1). Oysters collected on 7 June (60 d) exhibited significant mortality in the laboratory for 5 sites located within the inshore oyster farming areas (sites 14, 16, 23 and 37) or very close from them (site 39), suggesting that these animals were infected by OsHV-1 (File S3). Oysters deployed at these sites were the first to be hit by the mortality outbreak (Fig. 2), with the exception of oysters from site 16 (where *D*_0_ = 92 d). The oysters collected on 14 June and afterward (>67 d) showed significant mortalities in the laboratory, both for inshore farming sites and for several offshore sites located far away from the farms where no mortality was recorded on the field (e.g. sites 26, 28, 44). Interestingly, in laboratory conditions, oysters showed significant mortalities associated with OsHV-1 DNA detection (File S3) in 40 out of the 42 sites tested (95%), whereas only 15 sites (35%) were affected in the field. This reflects that (*i*) OsHV-1 can persist within an oyster without generating mortality in natural conditions (subclinical infection), and (*ii*) the spatial dispersion of the virus is much larger than the mortality pattern (Fig. 2).

### Spatial and temporal variations in environmental and host parameters

**Figure 3:**
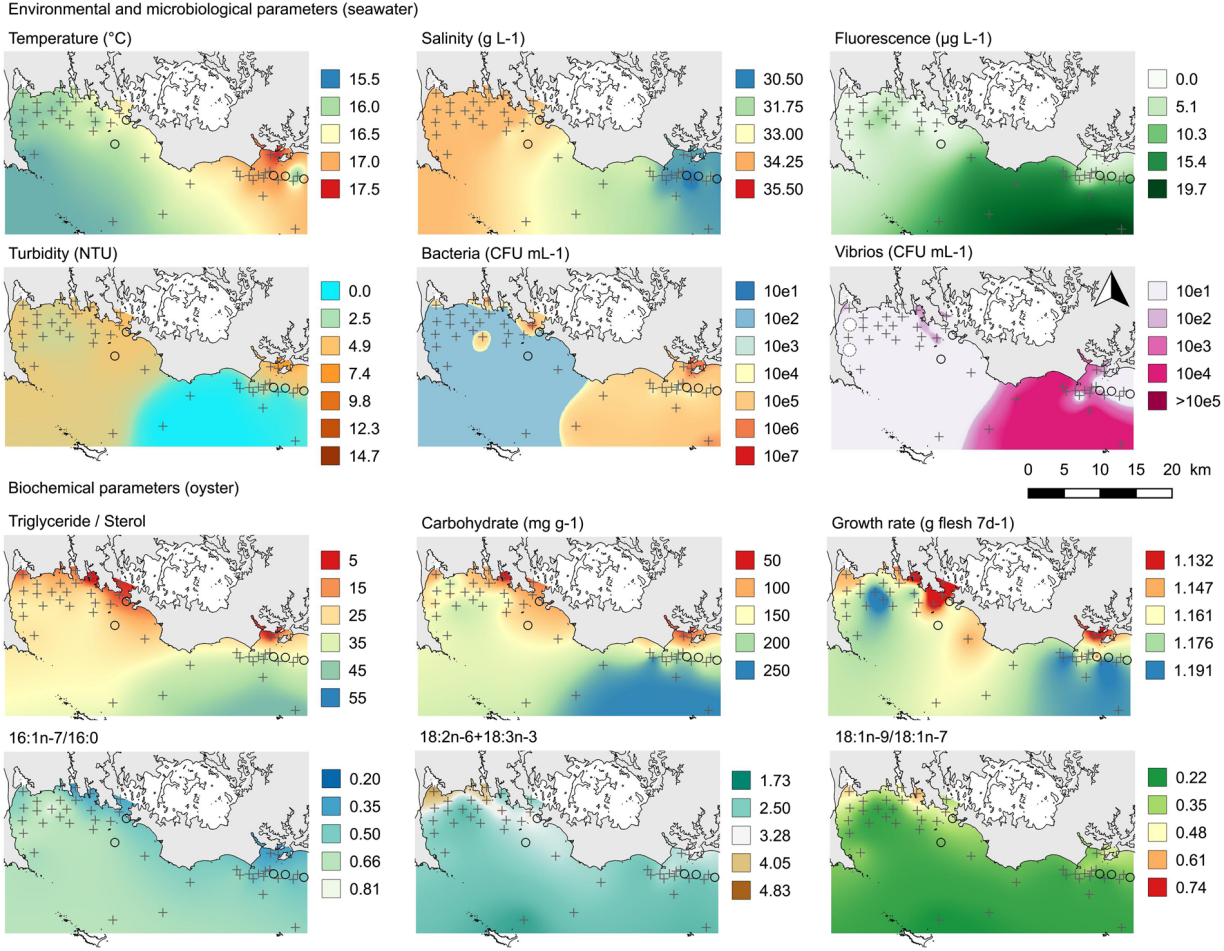
Environmental and host parameters on 20 June 2013 during the mortality event. The grey circles represent the sites where the sentinel oysters were accidentally lost and where only the seawater data are available. This map was done with the open source QGIS ver. 2.18.9. (QGIS Development Team, 2016, https://qgis.org).

The whole dataset of physical, microbiological, biochemical and biometrical parameters was mapped (File S7), and selected variables measured on the 20 June (73 d), during the mortality event are presented (Fig. 3). From 30 April to 25 July average seawater temperature increased from 11.2 °C to 20.0 °C, and decreased thereafter. There was a thermal gradient seaward, and the Penerf area appeared slightly warmer than the Quiberon Bay (Fig. 3). Salinity varied from 29.8‰ to 35.4‰ (Fig. S7.2). Chlorophyll-*a* fluorescence was generally higher in the Pernerf area than in the Quiberon Bay and peaked in the eastern part of the study area on 20 June (Fig. 3). This phytoplankton patch was characterized by low salinity, low turbidity and high concentration of vibrios in seawater (Fig. 3). Turbidity was generally low, typical of late spring and summer conditions (Fig. S7.4). Bacteria and vibrio concentrations in seawater were lower and less spatially structured than in oysters (Figs S7.6–S7 and 9).

On average, energy reserves of oysters decreased from 38% for carbohydrates to 51% for TAG between 30 April and 14 June (Figs S7.10 and S7.11), and gradually increased seaward thereafter. The spatial pattern of oyster energy reserves mirrored chlorophyll-*a* fluorescence on 20 June (Fig. 3). The levels of 16:1n-7/16:0, 20:5n-3/22:6n-3 and PUFA/SFA increased between 27 May and 20 June whereas 18:2n-6 + 18:3n-3, branched +15:0 + 17:0, and 18:1n-9/18:1n-7 decreased during this period. On 20 June, some of these indicators exhibited positive (16:1n-7/16:0) or negative (18:2n-6 + 18:3n-3, branched +15:0 + 17:0, and 18:1n-9/18:1n-7) gradients from the coast to the sea (Fig. S7.12). The spatial pattern of 16:1n-7/16:0 on 14 June mirrored oyster energy reserves and chlorophyll-*a* fluorescence on 20 June. Oyster growth rate (shell length, total body mass, shell mass and flesh mass) were the lowest along the coast and the highest where fluorescence peaked on 20 June (Fig. S7.13).

### Mortality risk factors of oysters

To better appraise the influence of the environment on the mortality event, the relationship between the mortality-free time (*D*_0_) and all environmental parameters was investigated using the Cox regression model (Table 2). Increasing bathymetry and physical distance to inshore oyster farms were associated with a lower risk of mortality (higher *D*_0_, Table 2), confirming the seaward spread of the disease outbreak. Two clusters of biogeochemical parameters were then observed: the environmental factors reducing the mortality risk (corresponding to negative estimate and higher *D*_0_), and those enhancing the mortality risk (corresponding to positive estimate and lower *D*_0_). In the first group, increasing oyster growth rate, energy reserves (TAG, TAG/ST and carbohydrates), contribution of diatoms to oyster diet (16:1n-7/16:0), food freshness (PUFA/SFA), chlorophyll-*a* fluorescence, oxygen content, seawater temperature and vibrio concentration in oyster were all associated with a lower risk of mortality (Table 2). In contrast, increasing turbidity, salinity and the contribution of animal (18:1n-9/18:1n-7), terrestrial (18:2n-6 + 18:3n-3) and bacterial fatty acids to the diet of oysters were associated with a higher risk of mortality (Table 2).

**Table 2:** Relationship between environmental, microbiological, biochemical and biometrical parameters and the mortality-free time (*D*_0_) of oysters by univariate Cox proportional hazards models.

N	Variable	Estimate	SE	χ^2^	p	Odds ratio
1	18:1n-9/18:1n-7_last_	0.117	0.020	32.91	<0.001	1.124
2	Distance to inshore oyster farms	−0.741	0.134	30.49	<0.001	0.476
3	Turbidity_mean_	0.229	0.042	30.21	<0.001	1.257
4	16:1n-7/16:0_mean_	−0.180	0.035	26.78	<0.001	0.835
5	TAG/ST_last_	−0.685	0.140	23.91	<0.001	0.504
6	Bathymetry	−0.324	0.069	22.01	<0.001	0.723
7	PUFA/SFA_last_	−0.035	0.008	20.45	<0.001	0.966
8	Flesh mass	−0.400	0.091	19.54	<0.001	0.670
9	Branched +15:0 + 17:0_last_	2.512	0.569	19.47	<0.001	12.326
10	Turbidity_last_	0.373	0.086	18.91	<0.001	1.451
11	TAG_last_	−0.101	0.024	18.23	<0.001	0.904
12	Fluorescence_last_	−0.174	0.041	18.00	<0.001	0.840
13	Shell mass	−0.246	0.059	17.33	<0.001	0.782
14	Salinity_last_	0.544	0.131	17.18	<0.001	1.722
15	PUFA/SFA_mean_	−0.056	0.014	16.84	<0.001	0.945
16	Bacteria in seawater_last_	0.000	0.000	16.75	<0.001	1.000
17	Total body mass	−0.285	0.070	16.54	<0.001	0.752
18	Fluorescence_mean_	−0.655	0.164	15.92	<0.001	0.520
19	16:1n-7/16:0_last_	−0.103	0.027	14.21	<0.001	0.902
20	Oxygen_last_	−0.083	0.023	12.85	<0.001	0.920
21	18:2n-6 + 18:3n-3_last_	1.385	0.395	12.33	<0.001	3.997
22	Carbohydrate _last_	−0.014	0.004	12.14	<0.001	0.986
23	20:5n-3/22:6n-3_mean_	0.032	0.009	11.61	0.001	1.032
24	Branched + 15:0 + 17:0_mean_	3.798	1.210	9.84	0.002	44.606
25	Shell length	−0.044	0.014	9.48	0.002	0.957
26	Temperature_mean_	−0.799	0.269	8.84	0.003	0.450
27	TAG/ST_mean_	−0.519	0.191	7.38	0.007	0.595
28	Vibrio in oyster_mean_	−0.192	0.071	7.21	0.007	0.825
29	Carbohydrate_mean_	−0.017	0.007	6.28	0.012	0.984
30	18:1n-9/18:1n-7_mean_	0.080	0.032	6.14	0.013	1.083
31	TAG_mean_	−0.071	0.032	4.82	0.028	0.931

When a variable was acquired more than once between deployment and *D*_0_, mean value (mean) and the value recorded before the onset of mortality (last) were used, except for the biometrical parameters where the overall growth rates were used. Only the significant parameters are presented and sorted in ascending order of χ^2^. Values of 18:1n-9/18:1n-7, 16:1n-7/16:0, PUFA/SFA and 20:5n-3/22:6n-3 were multiplied by 100 and concentrations of bacteria and vibrio in seawater and oysters were divided by 1000 to obtain manageable odds ratio.

**Figure 4:**
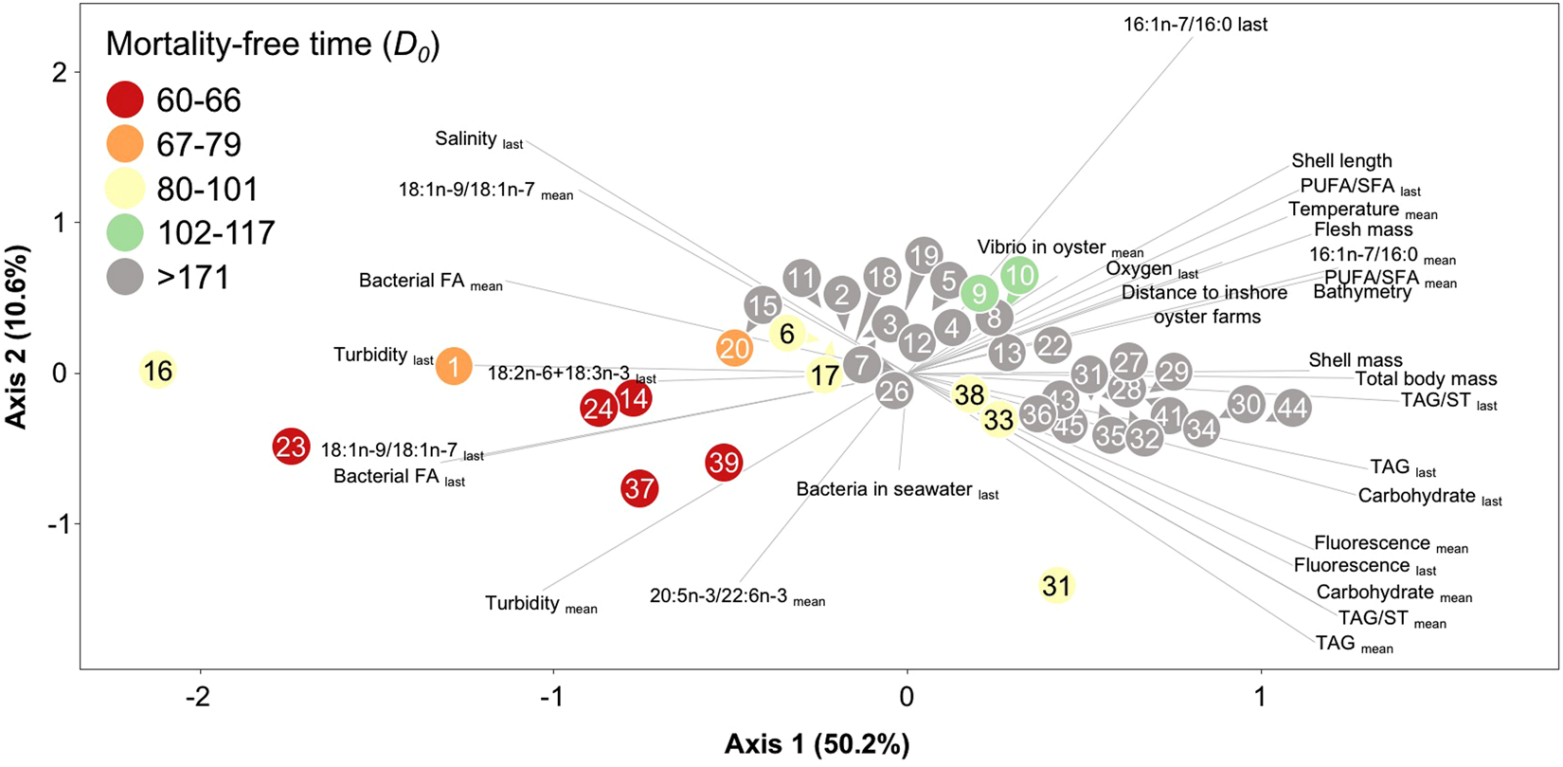
Principal component analysis of the parameters associated with mortality free-time of oysters (*D*_0_). Circles represent site numbers. See Table 2 for the list of parameters.

The principal component analysis of the environmental parameters associated with *D*_0_ confirms the classification in two groups. Most of the variance among sites (61%) was explained by the first two components, and all parameters associated with higher risk (lower *D*_0_) were located on the right side of the axis 1 while those related with lower risk were on the left side (Fig. 4).

The daily survival of oysters during the mortality event (β) and the duration of the mortality event (δ), two parameters describing the dynamics of mortality at the local scale (oyster bag), were not correlated with the environmental, biochemical and biometrical parameters (Table 3).

**Table 3:** Relationship between the daily survival of oysters during the mortality event (β) and the duration of the mortality event (δ) with environmental parameters.

Parameter	Descriptor	β		Δ	
		r	p	R	p
*Environment*					
Bathymetry	point	0.246	0.376	0.368	0.177
Distance to inshore oyster farms	point	0.164	0.559	0.161	0.567
Temperature	mean	0.171	0.541	0.236	0.398
	last	0.132	0.639	0.075	0.791
Salinity	mean	0.136	0.630	−0.221	0.428
	last	0.332	0.227	0.239	0.390
Fluorescence	mean	0.325	0.237	0.318	0.248
	last	0.457	0.087	0.521	0.056
Turbidity	mean	−0.361	0.187	−0.300	0.277
	last	−0.075	0.791	−0.114	0.685
Oxygen	mean	0.064	0.820	−0.079	0.781
	last	0.068	0.810	0.125	0.657
*Microbiology*					
Bacteria in seawater	mean	−0.461	0.084	−0.425	0.114
	last	−0.032	0.909	0.057	0.840
Vibrio in seawater	mean	−0.425	0.114	−0.517	0.049
	last	−0.186	0.507	−0.347	0.205
Bacteria in oyster	mean	−0.211	0.451	−0.204	0.467
	last	−0.532	0.041	−0.493	0.062
Vibrio in oyster	mean	−0.057	0.840	−0.089	0.752
	last	−0.418	0.121	−0.336	0.221
*Biochemistry*					
Carbohydrate	mean	0.211	0.451	0.261	0.348
	last	0.243	0.383	0.318	0.248
TAG	mean	0.046	0.870	0.150	0.594
	last	0.029	0.920	0.114	0.685
TAG/Sterol	mean	0.071	0.800	0.182	0.516
	last	0.029	0.920	0.118	0.676
16:1n-7/16:0	mean	0.308	0.265	0.309	0.262
	last	0.292	0.291	0.245	0.378
20:5n-3/22:6n-3	mean	0.214	0.443	0.111	0.695
	last	0.359	0.189	0.273	0.324
18:2n-6 + 18:3n-3	mean	0.154	0.585	0.175	0.533
	last	0.114	0.685	0.050	0.859
18:1n-9/18:1n-7	mean	0.047	0.869	0.055	0.844
	last	−0.111	0.695	−0.168	0.550
PUFA/SFA	mean	0.063	0.825	0.209	0.454
	last	0.057	0.840	0.120	0.671
Branched +15:0 + 17:0	mean	−0.246	0.376	−0.286	0.302
	last	−0.240	0.390	−0.316	0.251
*Biometry*					
Shell length	e^(b1)^	0.011	0.970	0.100	0.723
Total body mass	e^(b1)^	−0.161	0.567	−0.025	0.930
Shell mass	e^(b1)^	−0.171	0.541	−0.043	0.880
Flesh mass	e^(b1)^	0.032	0.910	0.104	0.713

Spearman correlation coefficients (r) and p-values are reported. When a variable was acquired more than once between deployment and *D*_0_, mean value (mean) and the value recorded before the onset of mortality (last) were used, except for the biometrical parameters where the overall growth rates were used.

## Discussion

Oyster disease broke out in the intertidal farming area when seawater temperature reached 16 °C and gradually spread seaward. Similarly, in the Mediterranean Thau lagoon, OsHV-1 induced mortalities started within the bivalve farms and spread outward^22,23^. These studies, conducted in contrasting environments at different spatial scales, indicate that conditions of high-density marine aquaculture (with Pacific oysters used here as an example), provides suitable conditions for disease epizootics^4^. However, wild oysters coexist with the farmed animals and they probably contributed to the disease outbreak. Wild oysters are often asymptomatic carrier of the virus capable of viral shedding^17^.

Mortalities and OsHV-1 DNA detection were observed in oysters placed 2 km from the nearest farming areas, but laboratory challenge revealed that oysters collected at nearly all sites were subclinically infected. Virus particles likely spread seaward via water currents since there was no other known source of OsHV-1 offshore. The oyster *C. gigas* is the only known source of OsHV-1, and this species is restricted to intertidal and shallow subtidal areas^9^. Although OsHV-1 DNA can be detected in other bivalve species, their role as vectors has not been demonstrated e.g.^24,25^. Therefore, OsHV-1 may travel over long distances, transported by seawater currents, reflecting openness and connectivity of the studied ecosystem and persistence of infective particles in seawater^4^. Considering that (*i*) average mean and maximum velocities of seaward current in the Mor-Braz area were respectively 0.15 m.s^−1^ and 0.43 m.s^−1^ (S. Petton pers. Comm.) and (*ii*) herpesviruses can persist for at least 24 h in seawater^26,27^, viral particles could travel 13–38 km before infecting a new host which is consistent with our results.

Subclinical infection of oysters at offshore sites may reflect (*i*) the dilution of viral particles below a threshold value under which no mortality occurs, and (*ii*) the low density of susceptible host. In line with this, mortality risk of oysters injected with viral suspension increases with concentration of OsHV-1 particles^28^. Also, mortality risk increases with the biomass of infected oysters and decreases with seawater renewal, two parameters that influence viral particles concentration^17^. Moreover, mortality risk of infected oysters may also be associated with spatial variations in overall environmental and host conditions.

Increasing chlorophyll-*a* fluorescence, contribution of diatoms to oyster diet (16:1n-7/16:0), food freshness (PUFA/SFA), oyster growth rate and energy reserves (triglycerides and carbohydrates) were all associated with a lower risk of mortality offshore. Aquatic consumers generally show increasing growth and survival performance with increasing dietary long-chain essential fatty acid content^29,30^. Also, energy balance influences immune functions, and consequently disease susceptibility in terrestrial invertebrates^31^. Therefore, well-fed oysters with high energetic reserves may be able to mount an efficient immune response and decrease mortality risk^23^.

Reciprocally, contribution of terrestrial (18:2n-6 + 18:3n-3), animal (18:1n-9/18:1n-7), and bacterial (branched + 15:0 + 17:0) organic matter to the food of oysters were associated with a higher risk of mortality. These items are secondary food sources associated with reduced growth rate of oysters^32^. Low food quality may reduce accumulation of energy reserves which in turn alters immune response and disease susceptibility of oyster.

Increasing turbidity, an optical proxy of the concentration of suspended particulate matter (SPM), was associated with a higher risk of OsHV-1 related mortality. Several studies show that SPM facilitate disease transmission by providing microhabitats for viruses^12,33^. Like other marine viruses, OsHV-1 may adsorb and bind to both organic and inorganic particles, so that turbidity is a mortality risk factor for oysters. Alternatively, turbidity may increase mortality risk of oysters by attenuating the penetration of ultraviolet (UV) irradiance in the water column. Indeed, UV radiation decrease infectivity of bacteriophages and viruses infecting eukaryotic cells^11,12^, and they inactivate OsHV-1 in controlled conditions^34^. Finally, turbidity correlated positively with terrestrial inputs inferred from the fatty acid composition of oysters and negatively with chlorophyll-*a* fluorescence. Therefore, turbidity may reflect particles of poor nutritional quality (i.e. inorganic particles), that reduced feeding efficiency and energy intake of oysters^9^.

Oyster mortality initiated when seawater temperature reached 16 °C, confirming that temperature is a triggering risk factor^35,36^. However, at offshore sites, oyster mortalities started later when temperature was above 19 °C. This explains that the temperature measured before the onset of mortality cannot be associated with mortality risk in this study.

Apparently paradoxical results may reflect spurious correlations due to confounding factors. For instance, increasing salinity between 29.8 and 35.4‰ was associated with a higher risk of mortality whereas survival of oysters exposed to OsHV-1 at 35‰ is ∼20% higher than at 25‰ in laboratory experiment^37^. Also, increasing concentrations of vibrios in oysters were associated with a lower mortality risk while vibrios are involved in disease development^38^. Low salinity and high concentration of vibrios were confounded by distance to inshore oyster farms, food quantity and quality, growth rate of oyster and their energy reserve so that they may not be causal risk factors.

The daily survival of oysters during the mortality event (β) and the duration of the mortality event (δ), two parameters describing the dynamics of mortality at the bag scale, were not correlated with any of the studied parameters. Environmental and host parameters influence the spatio-temporal dynamics of mortality at the regional scale (*D*_0_), but not at the local scale (β and δ). Mortality risk in an oyster bag may depend on different factors acting on small spatial scales, including the biomass of susceptible hosts and the concentration of OsHV-1 particles^17^.

Oysters kept offshore were exposed to OsHV-1 but showed no abnormal mortality. Therefore, offshore aquaculture, a considered option to face the increasing demand for space on the intertidal zone, has an epidemiological advantage^39^. Nevertheless, care must be taken to (*i*) not recreate farming conditions (host density) that are similar to those prevailing inshore, (*ii*) limit the import of oysters to certified animals without OsHV-1 in order to maintain viral load to natural background and (*iii*) evaluate the economic and social costs of moving offshore.

However, the oysters kept offshore were subclinically infected. This reveals that the spatial distribution of the virus was much wider than that of oyster mortality. Also, these infected animals could exhibit mortality once environmental and host conditions permit virus replication, so that the risk of disease transmission can increase. The laboratory challenge supports this hypothesis by revealing asymptomatic carriers of OsHV-1, and is complementary to qPCR assays for characterizing the health status of oysters.

Overall, oyster health was associated with indicators of good ecological status of the coastal environment. Indeed, the mortality risk of oysters was maximal in inshore and intertidal farming areas with high oyster biomass, and decreases with distance to inshore oyster farms, food quantity and quality (diatoms). Reciprocally, the risk increased with turbidity, terrestrial inputs and poor food quality (terrestrial, animal and bacterial sources). Therefore, limiting the impact of disease by maintaining good ecological status of coastal waters is likely an alternative to traditional disease management strategies such as culling, vaccination, and chemotherapy that are proven inefficient for most marine invertebrates^4^.

Several risk factors identified in this study can be mapped using GIS and ocean color satellite remote sensing. Turbidity can be quantified in coastal waters using high resolution satellite observations, and remote sensing of colored dissolved organic matter can be used as a proxy of terrestrial inputs. By providing a spatially explicit framework in which OsHV-1 mortality risk factors would be predicted from GIS (distance to inshore oyster farms and bathymetry) and satellite remote sensing (turbidity and terrestrial inputs), our study opens original perspective in the application of Earth Observation for the sustainable management of oyster farming ecosystems.

## Electronic supplementary material

Supplementary information
