# Supplementary material for: Determination of risk factors for herpesvirus outbreak in oysters using a broad-scale spatial epidemiology framework
Source: Sci Rep. 2018 Jul 18;8:10869. doi: 10.1038/s41598-018-29238-4 (PMC6052024; doi:10.1038/s41598-018-29238-4)
Supplement: Supplementary file 1 — Supplementary information [file 41598_2018_29238_MOESM1_ESM.docx]

**Determination of risk factors for herpesvirus outbreak in oysters using a broad-scale spatial epidemiology framework**

**Fabrice Pernet^1*^, Marine Fuhrmann^1^, Bruno Petton^2^, Joseph Mazurié^3^, Jean-François Bouget^3^, Elodie Fleury^1^, Gaétan Daigle^4^ and Pierre Gernez^5^**

^1^ Ifremer, Unité de Physiologie Fonctionnelle des Organisme Marins, LEMAR UMR 6539, Technopole de Brest-Iroise, Plouzané, France

^2^ Ifremer, Unité de Physiologie Fonctionnelle des Organisme Marins, LEMAR UMR 6539, Presqu’île du vivier, Argenton, France

^3^ Ifremer, Unité Littorale, Laboratoire Environnement Ressource du Morbihan Pays-de-la-Loire, 12 Rue des Résistants, La Trinité-sur-Mer, France

^4^ Université Laval, Département de mathématiques et de statistique, Pavillon Alexandre-Vachon, Québec, QC, Canada

^5^ Mer Molécules Santé (EA 2160), Université de Nantes, Nantes, France

**^*^** [fabrice.pernet@ifremer.fr](mailto:fabrice.pernet@ifremer.fr)

**Supplementary information**

**File S1. Animals and experimental set-up p2**

**File S2. Laboratory analyses p5**

**File S3. Laboratory challenge p8**

**File S4. Observatory network data p13**

**File S5. Survival analyses p15**

# **File S6. Biometrical analyses p18**

# **File S7. Maps of environmental and host parameters p23**

# **References p39**

# **File S1. Animals and experimental set-up**

Wild oyster spats were collected in Fouras (Marennes-Oléron, France) in 2010 and were moved to grow-up areas located at Paimpol and at Aber Benoît (Brittany, France) in 2011. These animals were exposed to diseases during the spring 2011 and suffered mortality >50%. In July 2012, 60 individuals were transferred to the experimental Ifremer facilities located at Argenton (48° 48’ 24.49” N, 3° 0’ 22.84” W) and treated by chloramphenicol (8mg/L) for 5 days prior to maturation conditioning. They were held at 17°C in 500 l flow-through tanks for conditioning. Fertilization occurred on September 2012 by stripping the gonads. The embryos developed in 150 l tanks at 21°C for 48 h, and D-larvae were transferred to flow-through rearing systems at 25°C. After 15 days, competent larvae were allowed to settle in downwellers. These rearing procedures were conducted in UV-sterilized, 1-µm-filtered seawater enriched with a mixture of *Chaetoceros muelleri* (CCAP 1010/3) and *Tisochrysis lutea* (CCAP 927/14) (1:1 in dry weight).

When oysters were >2 mm shell length, they were transferred to the Ifremer nursery in Bouin (46°57′15.5″N 2°02′40.9″W). On Mid-March 2013, a subsample of the oyster population was exposed to a thermal elevation in the laboratory from <14 to 21°C to test for the presence of OsHV-1 ^1^. OsHV-1 DNA was not detected and no mortality occurred, so that these oysters were considered as naïve with regard to the virus. On April 2013, they were transferred at la Trinité-sur-Mer (47°35'28.4"N 3°01'44.9"W) and placed in mesh bags before deployment in the study area (mean wet weight = 0.51 g).

These oysters were deployed at 46 sites located in the Mor-Braz area (Figure 1, Table S1.1). At each site, 16 small mesh bags (30×25×2 cm, Ø=6.0 mm) containing 85 individual oysters were grouped in one big mesh bag (90×45×8 cm, Ø=10.0 mm). These bags were attached to iron table for the sites situated in the intertidal farming area or immersed vertically at 2 meters deep and attached to 5L-buoy tied up to a mooring point for the sites in the offshore area.

Table S1.1 Site number, position, rearing structure, presence of oyster farms, coordinates, and distance and name of the nearest inshore farm area for 46 survey sites in the Mor-Braz area, France.

| Site | Position | | Rearing structure | Oyster farming | Latitude (°N) | Longitude (°W) | Nearest inshore oyster farm area†† | |
| --- | --- | --- | --- | --- | --- | --- | --- | --- |
|  |  |  |  |  |  |  | Distance (km) | Name |
| 1 | Inshore | Intertidal | Table | Yes | 47.5684208 | -3.1113752 | -0.6 | Le Pô |
| 2 | Offshore | Subtidal | Mooring | Yes^*^ | 47.5479378 | -3.1078926 | 1.7 | Le Pô |
| 3 | Offshore | Subtidal | Mooring | Yes^*^ | 47.5320214 | -3.1089598 | 3.5 | Le Pô |
| 4 | Offshore | Subtidal | Mooring | Yes^*^ | 47.5164217 | -3.1052437 | 5.2 | Le Pô |
| 5 | Offshore | Subtidal | Mooring | No | 47.4931246 | -3.0827931 | 8.1 | Le Pô |
| 6 | Offshore | Subtidal | Mooring | Yes^*^ | 47.5577372 | -3.0842426 | 2.1 | Le Pô |
| 7 | Offshore | Subtidal | Mooring | Yes^*^ | 47.5456374 | -3.0830097 | 2.8 | Le Pô |
| 8 | Offshore | Subtidal | Mooring | Yes^*^ | 47.5309208 | -3.0703103 | 4.7 | Le Pô |
| 9 | Offshore | Subtidal | Mooring | Yes^*^ | 47.5489369 | -3.0557266 | 4.0 | Crac’h |
| 10 | Offshore | Subtidal | Mooring | Yes^*^ | 47.5399896 | -3.0399245 | 3.8 | Crac’h |
| 11 | Offshore | Subtidal | Mooring | Yes^*^ | 47.5608697 | -3.0414430 | 2.4 | Crac’h |
| 12 | Offshore | Subtidal | Mooring | Yes^*^ | 47.5502559 | -3.0282074 | 2.4 | Crac’h |
| 13 | Offshore | Subtidal | Mooring | No | 47.5328395 | -3.0212741 | 4.1 | Crac’h |
| 14 | Inshore | Intertidal | Table | Yes | 47.5780190 | -3.0144760 | -1.0 | Crac’h |
| 15 | Offshore | Subtidal | Mooring | No | 47.5621692 | -3.0126599 | 0.7 | Crac’h |
| 16 | Inshore | Intertidal | Table | Yes | 47.5718519 | -2.9866431 | -1.1 | St-Philibert |
| 17 | Offshore | Subtidal | Mooring | No | 47.5506689 | -2.9832773 | 1.2 | St-Philibert |
| 18 | Offshore | Subtidal | Mooring | No | 47.5391356 | -2.9741777 | 2.6 | St-Philibert |
| 19 | Offshore | Subtidal | Mooring | No | 47.5271525 | -2.9768781 | 3.9 | Morbihan |
| 20 | Offshore | Subtidal | Mooring | No | 47.5388851 | -2.9458279 | 2.7 | Morbihan |
| 21 | Offshore | Subtidal | Mooring | No | 47.5127686 | -2.9356123 | 4.9 | Morbihan |
| 22 | Offshore | Subtidal | Mooring | No | 47.4158527 | -2.9294995 | 15.5 | Morbihan |
| 23 | Inshore | Intertidal | Table | Yes | 47.5672010 | -2.9308103 | -1.5 | Morbihan |
| 24 | Offshore | Subtidal | Mooring | No | 47.5512346 | -2.9309443 | 1.0 | Morbihan |
| 25 | Offshore | Subtidal | Mooring | No | 47.5437012 | -2.9172780 | 1.3 | Morbihan |
| 26 | Offshore | Subtidal | Mooring | No | 47.4982511 | -2.8785468 | 7.1 | Morbihan |
| 27 | Offshore | Subtidal | Mooring | No | 47.4292340 | -2.8385171 | 15.3 | Morbihan |
| 28 | Offshore | Subtidal | Mooring | No | 47.4691829 | -2.7920161 | 11.1 | Morbihan |
| 29 | Offshore | Subtidal | Mooring | No | 47.4888645 | -2.7074505 | 4.4 | Penerf |
| 30 | Offshore | Subtidal | Mooring | No | 47.4824310 | -2.7011676 | 4.3 | Penerf |
| 31 | Offshore | Subtidal | Mooring | No | 47.4844307 | -2.6843511 | 3.1 | Penerf |
| 32 | Offshore | Subtidal | Mooring | No | 47.4787639 | -2.6764016 | 3.1 | Penerf |
| 33 | Offshore | Subtidal | Mooring | No | 47.4856637 | -2.6670514 | 2.1 | Penerf |
| 34 | Offshore | Subtidal | Mooring | No | 47.4599968 | -2.6564028 | 4.7 | Penerf |
| 35 | Offshore | Subtidal | Mooring | No | 47.4825135 | -2.6571184 | 2.2 | Penerf |
| 36 | Offshore | Subtidal | Mooring | No | 47.4893302 | -2.6549848 | 1.4 | Penerf |
| 37 | Inshore | Intertidal | Table | Yes | 47.5120300 | -2.6472838 | -1.2 | Penerf |
| 38 | Offshore | Subtidal | Mooring | Yes | 47.4913967 | -2.6465849 | 1.2 | Penerf |
| 39 | Inshore | Subtidal | Mooring | Yes† | 47.5107797 | -2.6329842 | 1.8 | Penerf |
| 40 | Offshore | Subtidal | Mooring | No | 47.4865465 | -2.6393852 | 1.9 | Penerf |
| 41 | Offshore | Subtidal | Mooring | No | 47.4811797 | -2.6302190 | 2.8 | Penerf |
| 42 | Offshore | Subtidal | Mooring | No | 47.4866461 | -2.6175356 | 3.1 | Penerf |
| 43 | Offshore | Subtidal | Mooring | No | 47.4810458 | -2.6024695 | 4.4 | Penerf |
| 44 | Offshore | Subtidal | Mooring | No | 47.4213453 | -2.5930226 | 10.0 | Penerf |
| 45 | Offshore | Subtidal | Mooring | No | 47.4876290 | -2.5950360 | 4.6 | Penerf |
| 46 | Offshore | Subtidal | Mooring | No | 47.4845954 | -2.5830362 | 5.5 | Penerf |

^*^ The oysters farming area is on the bottom.

† The sentinel oysters were 130 m away from the farming area.

†† The reference points used for distance calculations between site and the nearest inshore oyster farm area are represented on Fig. 1.

**File S2. Laboratory analyses**

BIOMETRY

Shell length was measured with a digital calliper (Mitutoyo, Paris, France) and wet mass was recorded with a Mettler precision balance (Mettler-Toledo, Viroflay, France).

MICROBIOLOGY

Cultivable bacteria and vibrio were quantified on oyster and seawater sampled at each site and time. Soft tissues of 10 oysters were pooled, weighted, and homogenized in sterile artificial seawater (10 mL g^-1^ of wet tissue) with a T25 digital Ultra-Turrax® fitted with sterile disposable dispersing element (IKA, Staufen, Germany). Then, 100µL of the tissue sample was spread on thiosulfate-citrate-bile salts-sucrose agar (TCBS, Difco Laboratories) in Petri dishes to quantify vibrios. Another sample of 100µL was diluted (1:100, v/v) and spread on marine broth medium to quantify cultivable bacteria. Seawater (100 µL) was directly plated on TCBS and marine broth media. The plates were incubated at 22°C for 2 d in TCBS and for 6 d in marine broth before counting the number of colony forming units (CFU mL^-1^ for seawater or CFU 100 mg^-1^ wet tissue for oysters).

Quantification of OsHV-1 DNA was conducted (1) on powder of 25 pooled oysters for animals directly sampled on the study area on 27 May, 20 June and 13 August or (2) on flesh of 5 pooled oysters homogenized in sterile seawater for animals collected on 19 July and further exposed to laboratory conditions at 21°C. In both cases, ground oyster tissues were diluted in artificial seawater (1:4 m/v). Total DNA was then extracted using a QIAgen QIAamp tissue mini kit according to the manufacturer’s protocol. The extracted DNA was stored at -20°C prior to pathogen detection and quantification by qPCR.

The detection and quantification of OsHV-1 DNA was carried out using a previously published real-time PCR protocol ^2^. This protocol used SYBR® Green chemistry with specific DPFor/DPRev primers targeting the region of the OsHV-1 genome predicted to encode a DNA polymerase catalytic subunit ^3^. Amplification reactions were performed using an Mx30005P real-time PCR thermocycler sequence detector (Stratagene, La Jolla, CA, USA) with 96-microwell plates ^2^. These analyses were performed by one approved laboratory (Laboratoire Départemental Vétérinaire de l’Hérault, Montpellier, France).

ENERGETIC RESERVES

Carbohydrates

Carbohydrates were quantified according to Dubois et al. ^4^. Powder aliquots (100 mg) were placed in Eppendorf tubes containing 1.5 mL nanopure water, homogenised for ~30 s with a T10 basic ultra Turrax (IKA, Staufen, Germany). A subsample of the diluted powder was mixed with a phenol solution (5% m/v) and 2.5 mL H_2_SO_4_ and incubated for twenty minutes ^4^. This subsample was then placed in a UV/VIS spectrophotometer and its absorbance measured at 490 nm. Total carbohydrate concentration was calculated using a standard calibration curve made with glucose and expressed in mg g^-1^ dry mass of tissues.

Lipids

Powder aliquots (300 mg) were placed in amber glass vials filled with 6 ml chloroform-methanol (2:1 v/v) and stored at -20 °C until use. Just before the analysis, the samples were sonicated for 5 min and centrifuged for 2 min at 1000 rpm. Neutral lipid classes were analysed by HPTLC (high performance thin layer chromatography) using a CAMAG system (Chromacim SAS, Moirans, France), consisting of a sampler (TLC Sampler 4) and a reader (TLC Scanner 3). Silica plates (HPTLC plates silica gel 60, 10*20 cm, Merck) previously conditioned with a 1:1 hexane-diethyl ether mixture were activated for 30 minutes at 120 °C. Lipid classes were separated using hexane-diethyl ether-acetic acid (20:5:0.5, v/v/v) followed by hexane-diethyl ether (97:3, v/v). The lipid classes appeared as black marks after the silica plates had been soaked in a solution of copper sulphate (3 %) - orthophosphoric acid (8 %) and heated to 120 °C for 20 minutes. To quantify the lipid classes, a mixture of standards was deposited on each plate. This mixture of standards was prepared in similar proportions to those found in the neutral lipids of oysters: 58 % menhaden oil for the triglycerides, 14 % cholesterol for the sterols, 3 % 1-octadecanol for the alcohols, 10 % cholesterol palmitate for the sterol esters, 13 % 1-0 hexadecyl-2,3 dipalmitoyl-rac-glycerol for the glyceride ethers and 2 % stearic acid for the free fatty acids. Results were obtained by making readings at 370 nm and quantifying the marks with the Wincats program. Identified compounds were sterols (ST; μg mg^−1^ tissues) and triacylglycerol (TAG; μg mg^−1^ tissues). Since TAG are mainly reserve lipids and ST are structural constituents of cell membranes, we used the TAG-ST ratio as a proxy for the relative contribution of reserve to structure, as reported in larvae of marine invertebrates ^5^.

FATTY ACIDS

An aliquot of the chloroform-methanol (2:1 v/v) was evaporated to dryness and recovered with three 500 mL washings of CHCl_3_-MeOH (98:2 v/v). The samples were placed at the top of a silica gel microcolumn (30 × 5 mm internal diameter; Kieselgel; 70–230 mesh [Merck, Lyon, France]; previously heated to 450°C and deactivated with 5% water). The neutral lipids were eluted with 10 mL CHCl_3_-MeOH mixture (98:2 v/v). The polar lipids were recovered with 15 mL methanol. A known amount of 23:0 fatty acid was added as an internal standard. Lipids were transesterified with 10 wt% boron trifluoride-methanol ^6^ and analysed according to the method described by Marty et al. ^7^. The fatty acid methyl esters (FAME) were analysed in a gas chromatograph with an on-column injector, a DB-Wax (30 m × 0.25 mm; 0.25 μm film thickness) capillary column and a flame ionization detector. Hydrogen was used as the carrier gas. Only the fatty acids in the neutral lipids of the animals are presented here, since neutral lipids reflect the fatty acid profiles of food consumed and could reveal information about trophic sources, whereas fatty acids in the polar lipids are less sensitive to dietary changes ^8^.

**File S3. Laboratory challenge**

METHODS

The remaining oysters collected on 27 May, 7, 14, 20 and 27 June, and 4, 11 and 19 July were brought back to the Ifremer hatchery in Argenton and held 21°C for 12 days under laboratory conditions to reveal asymptomatic carriers of OsHV-1 ^1^. The oysters were placed in 5 L jars (one for each site) at an initial mean density of 42 ± 8 individuals per jar. Each jar was filled with UV filtered seawater and covered with aluminium foil. All the jars were placed in the same room with controlled air temperature (21°C). Seawater was renewed twice a day and a phytoplankton mixture (6000 μm^3^ μl^-1^ of *Chaetoceros muelleri* CCAP 1010/3 and *Tisochrysis lutea* CCAP 927/14 1:1 in dry weight) was added at each water renewal.

The oysters from each site were tested in the laboratory, except for the sites where significant mortalities had occurred in the field. When possible (i.e. from 27 June onwards), survival of 3-month-old specific pathogen free (SPF) oysters was monitored in 3 to 6 jars evenly displayed in the room to check for cross-infection among jars (controls). Dead animals were counted every 0.5 to 2 days in each jar. Nonparametric estimates of the survivor function were computed according to Kaplan & Meier ^9^. Survival time was measured as days from the onset of the experiment when oysters were brought back to the laboratory. The data were read as the number of dead animals within each jar on each time. Final survival was calculated and compared among sites and collection times (Table S3.1). The survival analysis in the laboratory covers only 42 out of the 46 sites in the end because some oyster bags and mooring system were accidentally lost during the study.

To verify that the mortality events observed under laboratory conditions were related to OsHV-1, the oysters collected on 19 July were sampled for OsHV-1 DNA detection analysis. Pools of five oysters were taken in jars where mortality occurred (n=15; sites 7, 8, 10, 15, 17, 24, 28, 31, 32, 34, 35, 36, 43, 44, 45, 46, SPF5-6) and in jars free of mortality (n=9; sites 2, 3, 5, 18, 26, SPF1-4) twice in time (day 3 and 12). Samples were immediately stored at -20°C until analysis. The detection and quantification of OsHV-1 DNA was carried out using a real-time PCR protocol ^2^ (File S2). OsHV-1 DNA was referred to as “detected” when it was > 100 copies mg^−1^ wet tissue at least once across the two analyses. The survival time curves of oysters were compared using the Cox regression model ^10^ after adjustment for the effect of OsHV-1 detection. OsHV-1 DNA detection in oysters was coded 0 if not detected, 1 for detected.

RESULTS

Survival of the sentinel oysters in the laboratory

The sentinel oysters collected on the field on 27 May (49 d) and maintained under laboratory conditions at 21°C for 12 d showed no significant mortality (Table S3.1). Their survival was between 96-100%. The survival of oysters collected on 7 June (60 d) at 5 sites out of 42 located within the intertidal oyster farming areas (sites 14, 16, 23 and 37) or very close from them (site 39) was lower than 75% in the laboratory. The oysters collected on 14 June and after (> 67 d) showed significant mortalities in the laboratory, both in the inshore sites within the oyster farming areas and in several offshore sites far from the farms (e.g. sites 26, 28, 44). Overall, the oysters from 40 sites showed significant mortalities in laboratory conditions, at least once during the period of study (Table S3.1). The collected oysters at a given site showed either (*i*) one period of high survival followed by one period of mortality outbreaks with no change (sites 1, 6, 11, 14, 16, 20, 23, 24, 37, 38, 39 and 46), (*ii*) several alternating periods of mortality outbreak and high survival (sites 3, 7, 8, 10, 13, 15, 18, 19, 26, 28, 29, 30, 31, 32, 33, 34, 35, 36, 41, 43, 44 and 45), (*iii*) only one mortality outbreak in the laboratory (sites 5, 9, 12, 22, and 27), (*iv*) massive mortalities except for one period (site 17) or (*v*) no mortality outbreak (sites 2 and 4). In contrast, control oysters (SPF not previously deployed on the field) showed no significant mortality in laboratory conditions, and OsHV-1 DNA was not detected in their tissues. Therefore, cross-infection among jars (sites) was very unlikely.

Relationship between oyster mortality and detection of OsHV-1 DNA

The final survival of oysters in laboratory conditions was on average 85.4% when OsHV-1 was not detected compared to only 31.6% when OsHV-1 DNA was detected (Fig. S3.1). Odds of mortality were 6.6 times higher when OsHV-1 DNA was detected than when it was not (Table S3.2). Therefore, the mortality of oysters observed in laboratory conditions was associated with the detection of OsHV-1 DNA.

Table S3.1. Final survival (%) and detection of OsHV-1 DNA in sentinel oysters deployed in the Mor-Braz area and maintained under laboratory conditions at 21°C for 12 days, according to the date of sampling (days post-deployment) on the field and the sites. Survival of specific pathogen free (SPF) oysters is also reported. OsHV-1 DNA was referred to as “detected” when it is > 100 copies mg^−1^ wet tissue. Survival ≤ 75% are indicated in **bold**.

|  |  | 27 May  (49 d) | 7 June  (60 d) | 14 June  (67 d) | 20 June  (73 d) | 27 June  (80 d) | 4 July  (87 d) | 11 July  (94 d) | 19 July  (102 d) | |
| --- | --- | --- | --- | --- | --- | --- | --- | --- | --- | --- |
| Site |  | Survival (%) | | | | | | | | OsHV-1 DNA |
| 1 |  | 100 | 100 | **58** | **53** | - | - | - | - | - |
| 2 |  | 100 | 100 | 100 | 100 | 94 | 82 | 100 | 100 | Not detected |
| 3 |  | - | 100 | **38** | 100 | 97 | **63** | 75 | 100 | Not detected |
| 4 |  | 100 | 94 | 80 | 93 | 98 | 94 | 97 | 89 | - |
| 5 |  | 100 | 100 | 100 | 79 | 95 | **74** | 100 | 100 | Not detected |
| 6 |  | 100 | 100 | 98 | **24** | **8** | **53** | - | - | - |
| 7 |  | 100 | 100 | 100 | 100 | **56** | 92 | 90 | **48** | Detected |
| 8 |  | 96 | 100 | 100 | 100 | **25** | **54** | 98 | **28** | Detected |
| 9 |  | 100 | 97 | 100 | **29** | 100 | 95 | 95 | 92 | - |
| 10 |  | 100 | 100 | **20** | 100 | **55** | - | 90 | **27** | Detected |
| 11 |  | 98 | 100 | 100 | 100 | 96 | 93 | **74** | **69** | - |
| 12 |  | 100 | 100 | **43** | 93 | 100 | 100 | - | 97 | - |
| 13 |  | 96 | 100 | **47** | **33** | 97 | **63** | **50** | 97 | - |
| 14 |  | 100 | **75** | **29** | - | - | - | - | - | - |
| 15 |  | 100 | 100 | **33** | 100 | 100 | 90 | 90 | **51** | Detected |
| 16 |  | 94 | **69** | **35** | **53** | - | **19** | - | - | - |
| 17 |  | 100 | 100 | **47** | **32** | **24** | **50** | 100 | **22** | Detected |
| 18 |  | 100 | 100 | **14** | **20** | **39** | 93 | 98 | 100 | Not detected |
| 19 |  | 100 | 100 | **40** | **15** | 100 | 95 | **14** | 88 | - |
| 20 |  | 96 | 100 | **20** | **17** | **21** | - | - | - | - |
| 22 |  | 97 | 100 | 98 | **9** | 100 | 87 | 96 | 95 | - |
| 23 |  | 100 | **37** | **51** | - | - | - | - | - | - |
| 24 |  | 100 | 100 | **16** | **22** | - | - | - | 76* | Not detected |
| 26 |  | 100 | 100 | **22** | **5** | **40** | 85 | 88 | 100 | Not detected |
| 27 |  | 97 | 100 | 100 | **63** | 98 | Lost | Lost | Lost | - |
| 28 |  | 100 | 100 | **40** | **34** | 85 | 97 | **38** | **58** | Detected |
| 29 |  | 100 | 100 | **63** | **69** | 98 | 90 | **40** | 97 | - |
| 30 |  | 94 | 100 | **38** | 100 | 100 | 95 | **17** | **69** | - |
| 31 |  | 100 | 100 | 100 | **16** | 98 | 100 | **44** | **48** | Detected |
| 32 |  | 97 | 100 | **15** | 100 | 100 | **64** | **46** | **66** | Not detected |
| 33 |  | 100 | 100 | **56** | **30** | 90 | 98 | - | - | - |
| 34 |  | 100 | 100 | **64** | **21** | 85 | 98 | **36** | **56** | Detected |
| 35 |  | 100 | 100 | 95 | **50** | 97 | 89 | **12** | **30** | Detected |
| 36 |  | 100 | 91 | **49** | 100 | 98 | 97 | **31** | **14** | Not detected |
| 37 |  | 100 | **75** | **51** | **62** | - | - | - | - | - |
| 38 |  | 100 | 100 | 100 | 97 | 98 | **69** | **12** | - | - |
| 39 |  | 100 | **68** | **14** | - | - | - | - | - | - |
| 40† |  | 100 | 100 | Lost | - | - | - | - | - | - |
| 41 |  | 100 | 98 | 100 | 100 | 95 | **2** | 97 | **60** | - |
| 43 |  | 98 | 100 | **71** | **47** | - | 77 | 98 | **29** | Not detected |
| 44 |  | 98 | 95 | **57** | 100 | 93 | 100 | **39** | 84 | Not detected |
| 45 |  | 98 | 100 | 100 | **14** | **64** | 96 | 87 | **41** | Detected |
| 46† |  | 100 | Lost | Lost | *100* | *100* | *98* | *100* | ***26*** | Detected |
| SPF1 |  | - | - | - | 100 | 100 | 100 | 100 | 100 | Not detected |
| SPF2 |  | - | - | - | - | 100 | 100 | 100 | 100 | Not detected |
| SPF3 |  | - | - | - | - | 100 | 100 | 100 | 100 | Not detected |
| SPF4 |  | - | - | - | - | - | - | - | 100 | Not detected |
| SPF5 |  | - | - | - | - | - | - | - | 76 | Not detected |
| SPF6 |  | - | - | - | - | - | - | - | 87 | Not detected |

* indicates that the tested oysters survived a mass mortality event on the field.

† indicates the early losses of the SPF oysters. The mooring deployed at sites 21, 25 and 42 were accidentally lost on 15 May. Also, the oyster bags deployed at sites 46 and 40 were lost on 7 June and 14 June respectively. Since the mooring system was still in place at site 46, new SPF oysters were added on 14 June. Survival data of the new SPF oysters are indicated in *italic*.


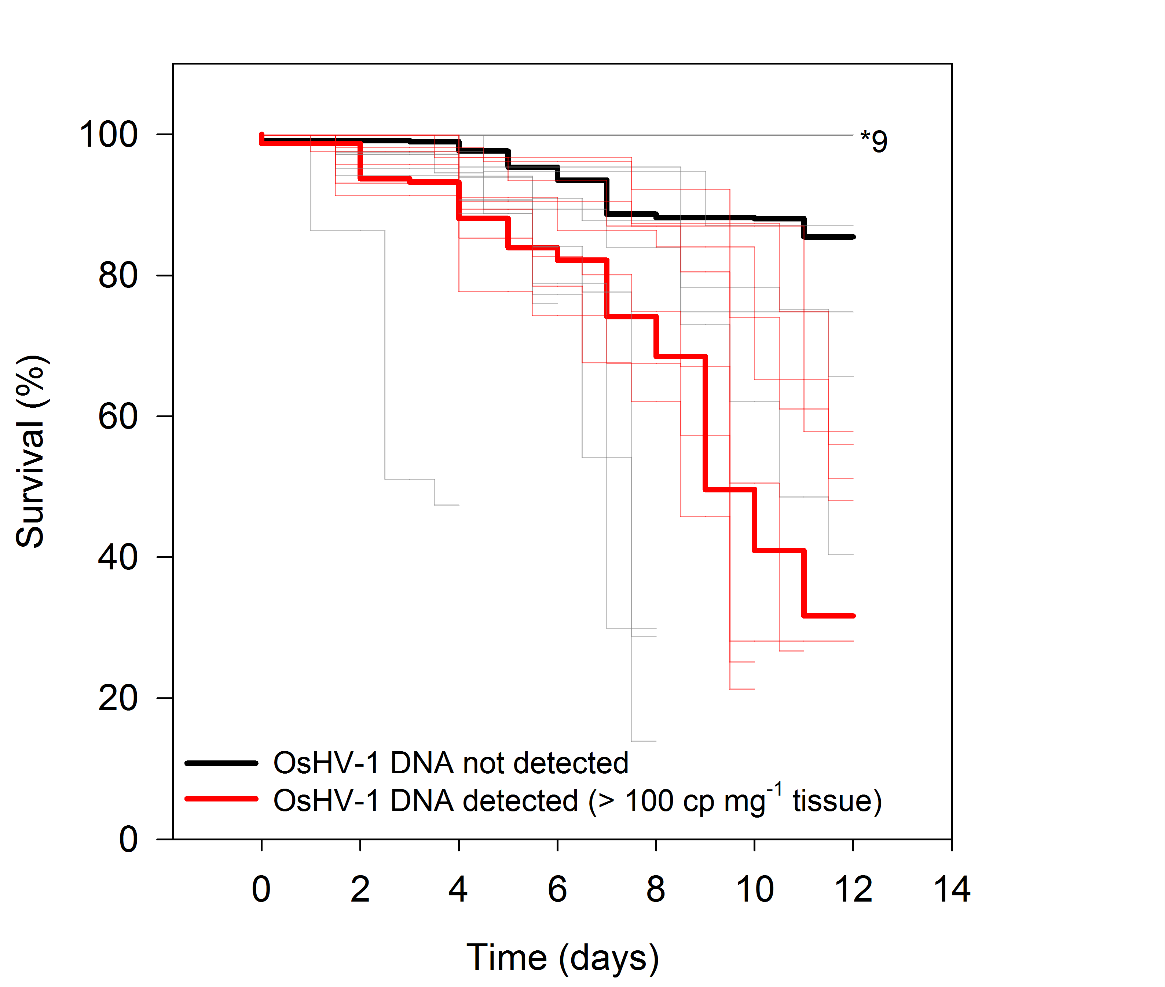
Figure S3.1 Survival of the sentinel oysters collected in the Mor-Braz area (n=21 sites) on 19 July and the specific pathogen free (SPF, n=6 jars) under laboratory conditions at 21°C for 12 days as a function of OsHV-1 DNA detection. OsHV-1 DNA was referred to as “detected” (red lines) when it is higher than 100 copies mg^−1^ wet tissue at least once across the two analyses, or not detected (black lines). Thin lines represent individual sites or batches of SPF oysters whereas bold lines represent the average survival curves of oysters negative (black) or positive (red) for the detection of OsHV-1 DNA. * indicates the overlap between 9 curves.

Table S3.2. Cox regression model examining the effect on survival of detection of OsHV-1 DNA in oysters. OsHV-1 DNA was referred to as “detected” when it is > 100 copies mg^−1^ wet tissue at least once across the two analyses.

| Source of variation | df | Estimate | SE | χ^2^ | p | Odds ratio |
| --- | --- | --- | --- | --- | --- | --- |
| Detection of OsHV-1 DNA | 1 | 1.89 | 0.13 | 225.78 | **< 0.001** | 6.60 |

**File S4. Observatory network data**

Since 2009, the shellfish observatory network monitored oyster mortality and pathogens along the French coastline (http://wwz.ifremer.fr/observatoire_conchylicole/). The data collected in 2013 at three sites in the farming area of the Mor-Braz are presented here. Two sites were in the intertidal zone (Pénerf and Larmor-Baden) and one was in the subtidal (Men-er-Roué, Table S4.1). Three-month-old oysters were produced by a private hatchery and transferred at la Trinité-sur-Mer on 10 March. Fifty oysters were screened for OsHV-1 DNA by qPCR and it was undetected at this time. On March 11, the oysters were placed in three mesh bags per site at a density of 350 individuals per bag (total biomass = 65.7 g per bag). The oyster bags were attached to iron tables for the intertidal sites or placed into a sea-cage in the subtidal site. Seawater temperature was measured every 15 minutes using temperature probes placed in one oyster bag at each site. Live and dead animals were counted twice a month during spring tides from April to December in one of the three bags. Three pools of three alive oysters were collected and analyzed for OsHV-1 DNA from early May to mid-September (one pool per bag).

Table S4.1 Regional location, position, bathymetry, rearing structure, site name, and coordinates for the 3 observatory sites in the Mor-Braz area, France.

| Region | Position |  | Bathymetry | Rearing structure | Site | Latitude (°N) | Longitude (°W) |
| --- | --- | --- | --- | --- | --- | --- | --- |
| Morbihan | Inshore | Intertidal | -1.01 | Table | Larmor-Baden | 47.592323 | -2.884589 |
| Quiberon | Offshore | Subtidal | 5.03 | Sea cage | Men-er-Roué | 47.538159 | -3.093013 |
| Pénerf | Inshore | Intertidal | -1.29 | Table | Pénerf | 47.510109 | -2.648004 |


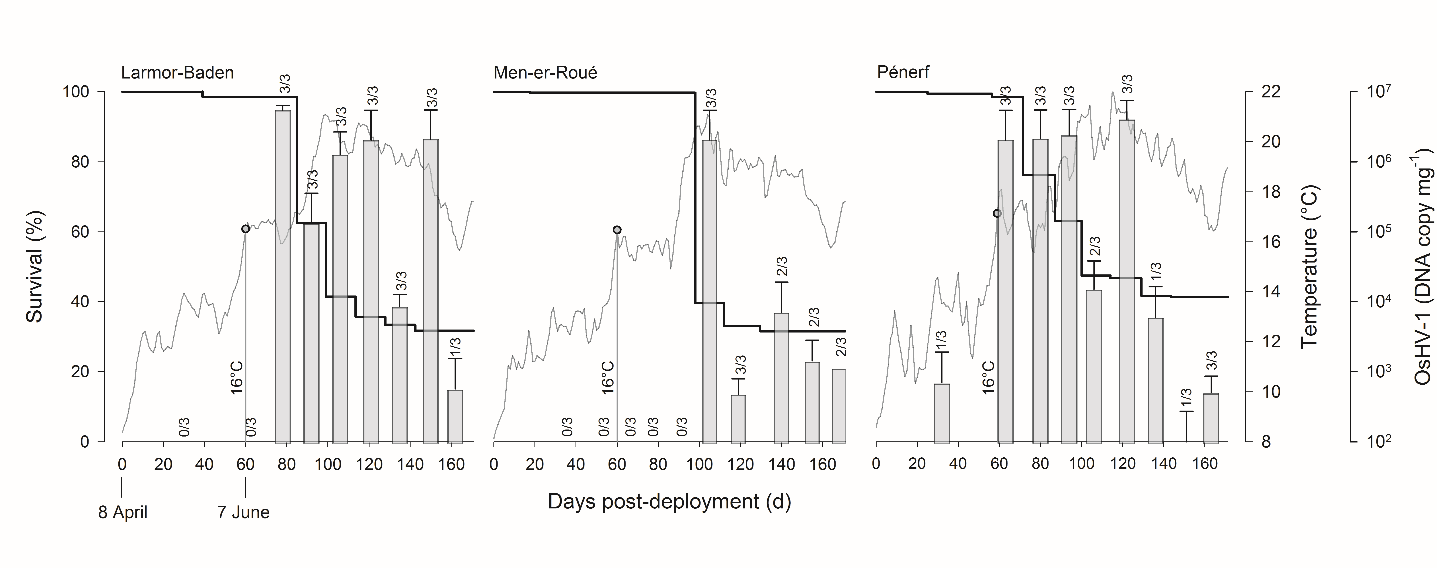
Figure S4.1 Survival of oysters at three sites in the Mor-Braz area. Left axis: survival functions of oysters. Right axis: evolution of seawater temperature and levels of OsHV-1 DNA in oyster tissues (mean ± s.d., n=3). Fractions indicate the number of positive samples out of the total number analyzed.

The oysters from the observatory network deployed in the Mor-Braz area were severely hit by the mass mortality phenomenon at the 3 sites (Fig. S4.1). Mortality first occurred at Pénerf between 10 June and 27 June (63 to 80 d after deployment of the sentinel oysters), while seawater temperature exceeded 16°C, and last until 8 August (122 d). This mortality event coincided with the detection of high levels of OsHV-1 DNA (>10^6^ cp mg g^-1^ wet tissue) in almost all the samples collected since 10 June (Fig. S4.1). The level of OsHV-1 DNA decreased below 10^4^ cp mg g^-1^ when the mortality had stabilized at *ca.* 42% on 22 August (136 d). In comparison, the temporal evolution of oyster mortality and of OsHV-1 DNA detection at the two other sites was delayed. Indeed, mortality and OsHV-1 DNA detection were first observed on 25 June (78 d) and 22 July (105 d) at Larmor-Baden and Men-er-Roué respectively. Also, the duration of the mortality event and the detection of OsHV-1 DNA in oysters was markedly reduced at Men-er-Roué.

**File S5. Survival analyses**

Survival of oyster was fitted a nonlinear regression model using the Marquardt algorithm ^11^ as a part of the NLIN procedure of the SAS software package (SAS 9.4, SAS institute, Carry, USA):

$$\left( Eq.1 \right) S=\left\{ \begin{aligned} \alpha\mathrm{if}d<D_{0} \\ \alpha+\beta(d-D_{0}) if D_{0}\leq d\leq D_{0}+\delta\\ \alpha+\beta\delta\mathrm{if}d>D_{0}+\delta\end{aligned} \right.$$

where *α* is the mean survival before the appearance of a mortality event, *d* is the number of days since the deployment of oyster on the field, *D_0_* is the number of days before the mortality event (also referred to as mortality-free time), β is the daily variation of survival of oysters during the mortality event, and δ is the duration of the mortality event (Figure S5.1).

Model parameters were estimated within the following range:

$\alpha\in\left[ 90, 100 \right] by$ 1

$$\beta\in\left[ -10,-0.5 \right] by 0.5$$

$$D_{0}\in\left[ 40, 95 \right] by 3$$

$\delta\in\left[ 10, 90 \right] by 5$.

Examples are presented on Figure S5.1 and model parameter estimates are listed on Table S5.1.


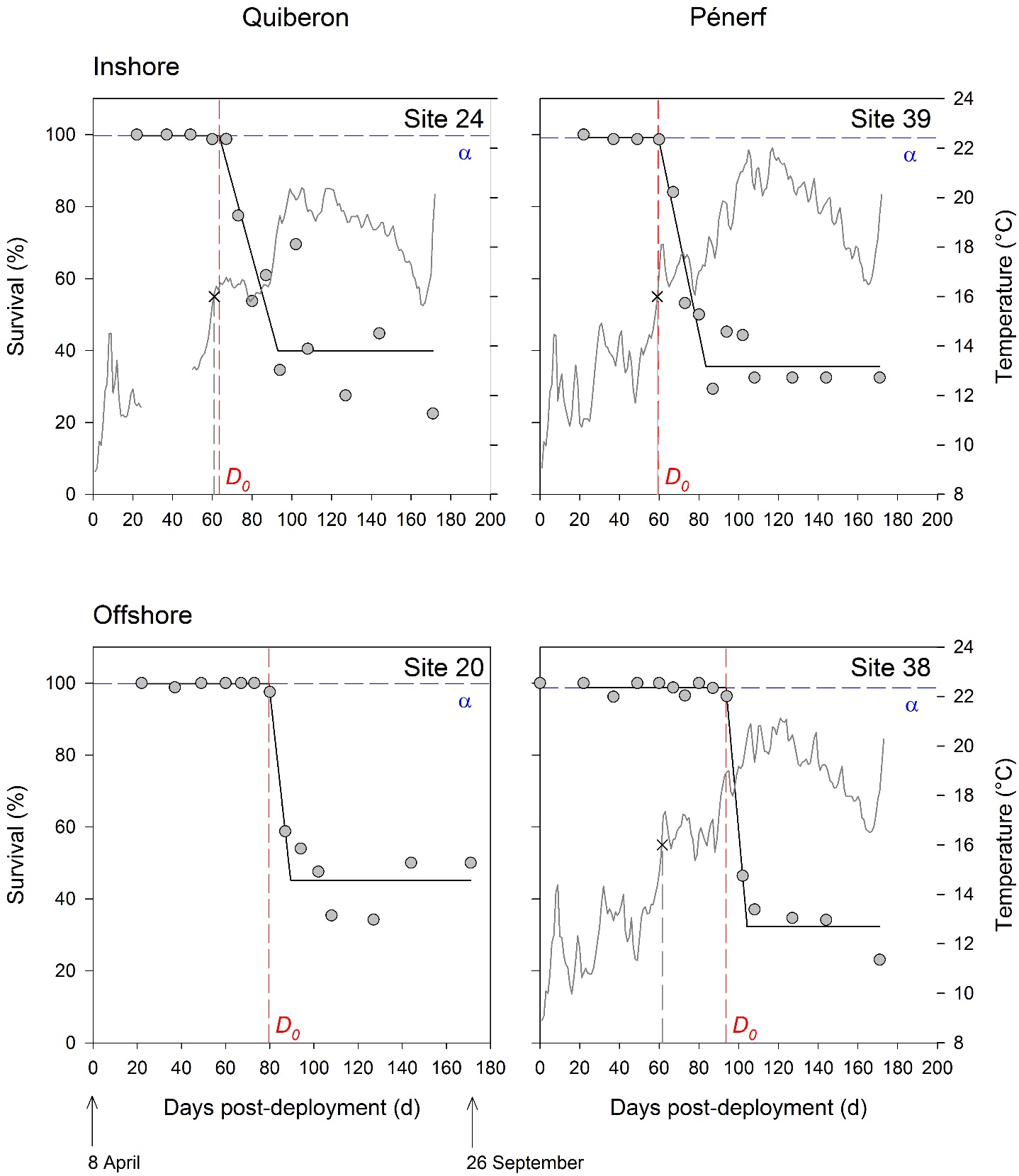


Figure S5.1 Observed (grey circles) versus simulated (black line) survival of oysters at four sites located inshore or offshore in the bay of Quiberon and in the Pénerf River. Value of α and *D_0_* are indicated by the dashed lines. Seawater temperatures are indicated where available (right axis). The threshold value of 16°C is indicated by a cross and reported on time axis (dashed grey line).

Table S5.1. Model parameter estimates for sites where mortality occurred. Abbreviations: *α* is the mean survival before the appearance of a mortality event, *D_0_* is the number of days before the mortality event, β is the daily variation of survival of oysters during the mortality event, and δ is the duration of the mortality event.

| Site | *α* (%) | *β* (% death d^-1^) | *D_0_* (d) | *δ* (d) |
| --- | --- | --- | --- | --- |
| 1 | 98.7 | -5.3 | 71.2 | 10.6 |
| 6 | 99.2 | -2.3 | 81.6 | 20.4 |
| 9 | 99.3 | -2.5 | 117.6 | 27.5 |
| 10 | 99.9 | -1.1 | 117.2 | 90.0 |
| 14 | 99.4 | -4.8 | 65.8 | 12.7 |
| 16 | 97.7 | -5.0 | 91.8 | 10.0 |
| 17 | 99.3 | -11.8 | 101.8 | 6.4 |
| 20 | 99.8 | -5.5 | 79.6 | 9.9 |
| 23 | 99.4 | -7.0 | 65.9 | 9.1 |
| 24 | 99.7 | -2.0 | 63.4 | 29.6 |
| 31 | 99.3 | -6.4 | 101.0 | 8.6 |
| 33 | 99.2 | -4.5 | 88.6 | 14.8 |
| 37 | 99.4 | -4.2 | 65.9 | 10.8 |
| 38 | 98.7 | -6.2 | 93.6 | 10.7 |
| 39 | 99.2 | -2.7 | 59.6 | 23.9 |
|  |  |  |  |  |
| Variable | Mean | SD | Min | Max |
| *α* | 99.2 | 0.5 | 97.7 | 99.9 |
| *D_0_* | 84.3 | 19.3 | 59.6 | 117.6 |
| *β* | -4.8 | 2.6 | -11.8 | -1.1 |
| *δ* | 19.6 | 20.8 | 6.4 | 90.0 |

**File S6. Biometrical analyses**

Biometrical measurements of oysters (shell length, total body mass, shell mass and flesh mass) were fitted using a segmented regression model according to the following equations:

$$\left( Eq. 2 \right) Y=\left\{ \begin{aligned} b_{0}+b_{1}\frac{d}{7} \mathrm{if} d<\hat{D_{0}} \\ b_{0}+b_{1}\frac{\hat{D_{0}}}{7}+b_{2}\frac{d-\hat{D_{0}}}{7} \mathrm{if} d\geq\hat{D_{0}} \end{aligned} \right.$$

Where *b_0_* is the intercept, *b_1_* and *b_2_* are the regression coefficients before and after the mortality event respectively, *d* is the number of days since the deployment of oyster on the field and $\hat{D_{0}}$ is the number of days before the mortality event (mortality-free time). For each biometrical parameter, a mixed segmented regression model was conducted to estimate parameters *b_1_* and *b_2_* while site was considered as a random factor. This was done using the MIXED procedure of the SAS software package (SAS 9.4, SAS institute, Carry, USA). The hypothesis of slope equality (H_0_: *b_1_*=*b_2_*) was tested using an *F-*statistic by mean of contrast (Table S6.1).

The regression coefficients *b_1_* and *b_2_* were different for shell length, shell mass and flesh mass but not for total body mass (Table S6.1, Figure S6.1). Overall shell length of oysters increased more rapidly after the onset of mortality than before. Indeed, their growth rate was 1.16 mm per week until mortality occurs compared to 1.46 mm afterward (Table S6.1). Total body mass of oysters increased at the same rate, irrespective of the mortality event. Therefore, a simple regression model with a constant slope was fitted to total body mass:

$$\left( Eq. 3 \right)\log(total body mass)=b_{0}+b_{1}\times\frac{d}{7},{\mathrm{and} b}_{1}=0.1465.$$

Shell mass of oysters increased less rapidly after the onset of mortality than before. The relative increase in shell mass was 16.1% per week (e^0.150^=1.161, Table S6.1) until mortality occurs compared to only 13.0% afterward (e^0.122^=1.130). Inversely, flesh mass of oysters increased more rapidly after the onset of mortality (16.4% per week, e^0.152^=1.164) than before (18.9% per week, e^0.173^=1.189).

Site specific regression coefficients are listed Table S6.2 for each biometrical parameter.

Table S6.1. Summary of the mixed model ANOVA for fixed effects and contrast between slopes for each biometrical parameter.

| Parameter | Coefficient^1^ | Estimate^2^ | Error | df | *t* | p > *t* | *F*-statistic  (*b_1_* vs. *b_2_*) | p > *F* |
| --- | --- | --- | --- | --- | --- | --- | --- | --- |
| Shell length | *b_0_* | 28.443 | 0.58 | 40 | 48.9 | **< 0.001** |  |  |
| (mm) | *b_1_* | 1.158 | 0.05 | 40 | 23.2 | **< 0.001** |  |  |
|  | *b_2_* | 1.459 | 0.07 | 39 | 21.3 | **< 0.001** | 12.9 | **< 0.001** |
|  |  |  |  |  |  |  |  |  |
| Total body | *b_0_* | 1.150 | 0.06 | 40 | 20.8 | **< 0.001** |  |  |
| mass | *b_1_* | 0.150 | 0.00 | 40 | 33.0 | **< 0.001** |  |  |
| (log g) | *b_2_* | 0.140 | 0.00 | 39 | 32.1 | **< 0.001** | 2.5 | 0.122 |
|  |  |  |  |  |  |  |  |  |
| Shell mass | *b_0_* | 0.819 | 0.05 | 40 | 16.1 | **< 0.001** |  |  |
| (log g) | *b_1_* | 0.150 | 0.00 | 40 | 33.3 | **< 0.001** |  |  |
|  | *b_2_* | 0.122 | 0.01 | 39 | 23.4 | **< 0.001** | 13.8 | **< 0.001** |
|  |  |  |  |  |  |  |  |  |
| Flesh mass | *b_0_* | -0.109 | 0.07 | 40 | -1.7 | 0.105 |  |  |
| (log g) | *b_1_* | 0.152 | 0.01 | 40 | 28.6 | **< 0.001** |  |  |
|  | *b_2_* | 0.173 | 0.01 | 39 | 31.2 | **< 0.001** | 8.6 | **0.006** |

^1^ Coefficient *b_0_* is the intercept, *b_1_* and *b_2_* are the regression coefficients before and after the mortality event respectively.

^2^ Estimates of *b_1_* and *b_2_* indicate the relative biometrical increase during one week, before and after the onset of mortality respectively. When data were log transformed, estimate needs to be exponentiated.


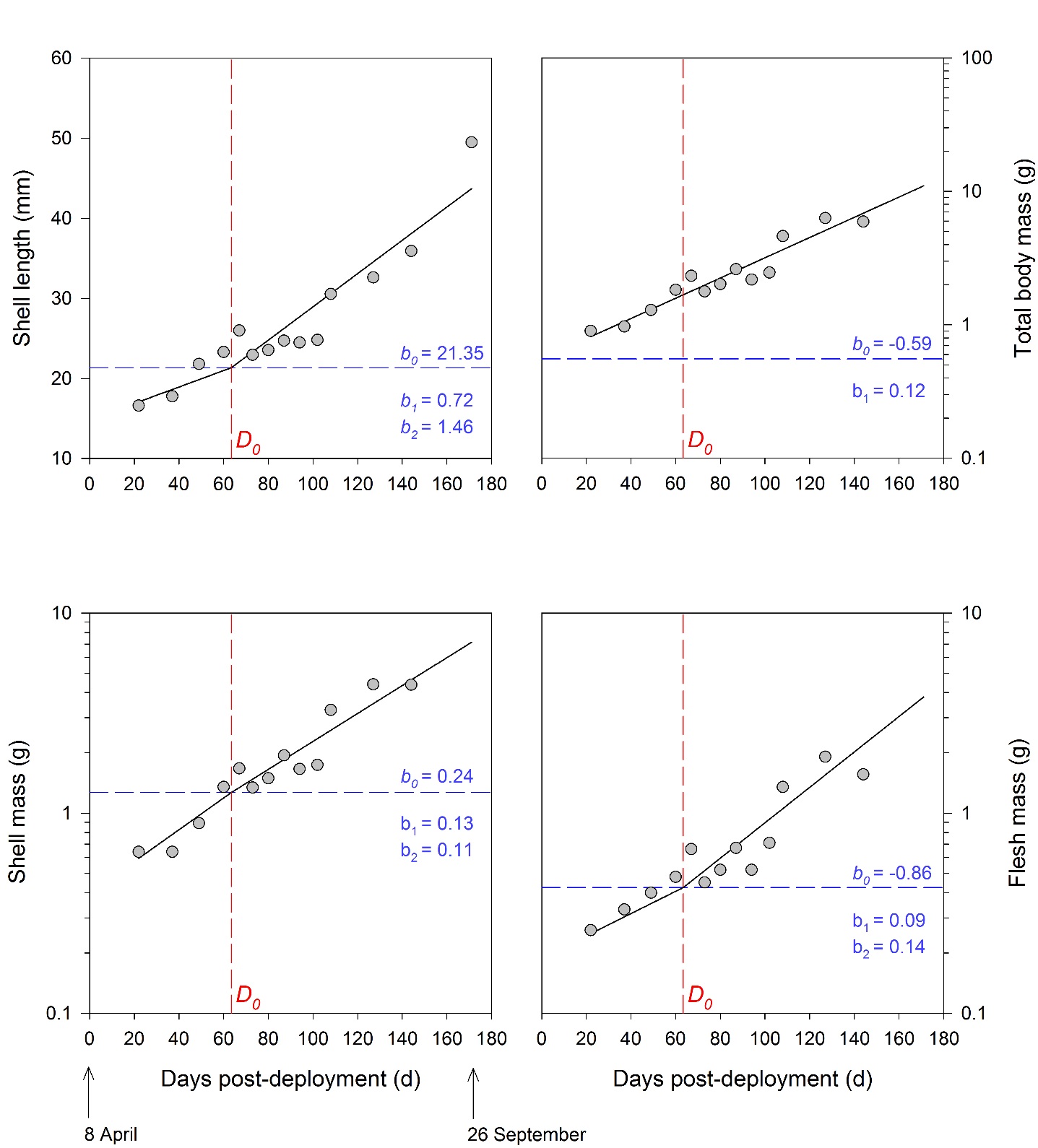
Figure S6.1 Observed (grey circles) versus simulated (black line) growth of oysters at site 24 located offshore in the bay of Quiberon. Values of $\hat{D_{0}}$ and *b_0_* are indicated by the dashed lines.

Table S6.2. Site specific regression coefficients for each biometrical parameter.

|  | Shell length | | |  | Total body mass (log) | |  | Shell mass (log) | | |  | Flesh mass (log) | | |
| --- | --- | --- | --- | --- | --- | --- | --- | --- | --- | --- | --- | --- | --- | --- |
| Site No | b_0_ | b_1_ | b_2_ |  | b_0_ | b_1_ |  | b_0_ | b_1_ | b_2_ |  | b_0_ | b_1_ | b_2_ |
| 1 | 23.50 | 0.78 | 1.10 |  | -0.62 | 0.13 |  | 0.39 | 0.14 | 0.12 |  | -0.62 | 0.14 | 0.17 |
| 2 | 29.77 | 1.15 | 1.08 |  | -0.64 | 0.15 |  | 0.88 | 0.16 | 0.11 |  | -0.10 | 0.14 | 0.18 |
| 3 | 27.77 | 1.14 | 1.55 |  | -0.64 | 0.14 |  | 0.76 | 0.15 | 0.13 |  | -0.19 | 0.16 | 0.17 |
| 4 | 27.74 | 1.12 | 1.56 |  | -0.65 | 0.15 |  | 0.83 | 0.15 | 0.12 |  | -0.13 | 0.16 | 0.18 |
| 5 | 29.39 | 1.26 | 1.70 |  | -0.66 | 0.16 |  | 0.90 | 0.15 | 0.13 |  | 0.02 | 0.16 | 0.17 |
| 6 | 27.36 | 1.03 | 1.11 |  | -0.62 | 0.14 |  | 0.71 | 0.14 | 0.11 |  | -0.27 | 0.14 | 0.17 |
| 7 | 29.42 | 1.21 | 1.39 |  | -0.63 | 0.15 |  | 0.84 | 0.13 | 0.12 |  | 0.03 | 0.16 | 0.15 |
| 8 | 29.98 | 1.25 | 1.55 |  | -0.66 | 0.15 |  | 0.88 | 0.16 | 0.12 |  | 0.01 | 0.17 | 0.18 |
| 9 | 36.77 | 1.58 | 0.79 |  | -0.65 | 0.15 |  | 1.44 | 0.15 | 0.13 |  | 0.80 | 0.19 | 0.17 |
| 10 | 38.84 | 1.85 | 1.39 |  | -0.65 | 0.15 |  | 1.43 | 0.15 | 0.13 |  | 0.85 | 0.19 | 0.17 |
| 11 | 28.39 | 1.17 | 1.52 |  | -0.63 | 0.14 |  | 0.68 | 0.14 | 0.13 |  | -0.18 | 0.16 | 0.17 |
| 12 | 27.48 | 1.16 | 1.82 |  | -0.64 | 0.14 |  | 0.65 | 0.15 | 0.13 |  | -0.16 | 0.15 | 0.18 |
| 13 | 28.47 | 1.21 | 1.85 |  | -0.66 | 0.15 |  | 0.77 | 0.16 | 0.14 |  | -0.10 | 0.16 | 0.19 |
| 14 | 24.31 | 0.83 | 1.08 |  | -0.61 | 0.13 |  | 0.40 | 0.14 | 0.07 |  | -0.64 | 0.13 | 0.16 |
| 15 | 29.35 | 1.24 | 1.64 |  | -0.65 | 0.15 |  | 0.93 | 0.16 | 0.12 |  | -0.14 | 0.14 | 0.19 |
| 16 | 21.07 | 0.64 | 0.88 |  | -0.48 | 0.07 |  | 0.12 | 0.07 | 0.11 |  | -0.93 | 0.10 | 0.11 |
| 17 | 32.64 | 1.31 | 0.98 |  | -0.65 | 0.15 |  | 1.26 | 0.16 | 0.14 |  | 0.32 | 0.17 | 0.18 |
| 18 | 29.35 | 1.24 | 1.67 |  | -0.65 | 0.15 |  | 0.89 | 0.16 | 0.13 |  | -0.10 | 0.15 | 0.18 |
| 19 | 28.28 | 1.23 | 1.80 |  | -0.64 | 0.15 |  | 0.79 | 0.14 | 0.13 |  | -0.17 | 0.15 | 0.17 |
| 20 | 26.62 | 1.05 | 1.47 |  | -0.61 | 0.13 |  | 0.67 | 0.15 | 0.11 |  | -0.48 | 0.11 | 0.17 |
| 22 | 27.75 | 1.15 | 1.65 |  | -0.64 | 0.14 |  | 0.73 | 0.15 | 0.13 |  | -0.19 | 0.15 | 0.17 |
| 23 | 23.39 | 0.74 | 0.86 |  | -0.59 | 0.12 |  | 0.28 | 0.12 | 0.11 |  | -0.88 | 0.11 | 0.15 |
| 24 | 21.35 | 0.72 | 1.46 |  | -0.59 | 0.12 |  | 0.24 | 0.13 | 0.11 |  | -0.86 | 0.09 | 0.14 |
| 26 | 27.25 | 1.17 | 1.75 |  | -0.62 | 0.14 |  | 0.70 | 0.15 | 0.13 |  | -0.30 | 0.13 | 0.17 |
| 27 | 28.79 | 1.18 | 1.51 |  | -0.65 | 0.15 |  | 0.83 | 0.14 | 0.12 |  | -0.06 | 0.15 | 0.17 |
| 28 | 26.80 | 1.12 | 1.83 |  | -0.64 | 0.14 |  | 0.71 | 0.15 | 0.13 |  | -0.14 | 0.16 | 0.18 |
| 29 | 29.50 | 1.29 | 1.78 |  | -0.68 | 0.16 |  | 0.96 | 0.16 | 0.14 |  | 0.08 | 0.17 | 0.18 |
| 30 | 28.61 | 1.23 | 1.78 |  | -0.68 | 0.16 |  | 0.91 | 0.16 | 0.15 |  | 0.07 | 0.18 | 0.18 |
| 31 | 33.76 | 1.48 | 1.31 |  | -0.66 | 0.16 |  | 1.37 | 0.16 | 0.11 |  | 0.53 | 0.17 | 0.17 |
| 32 | 30.40 | 1.29 | 1.65 |  | -0.69 | 0.17 |  | 1.13 | 0.19 | 0.12 |  | 0.19 | 0.16 | 0.21 |
| 33 | 29.13 | 1.09 | 0.89 |  | -0.63 | 0.15 |  | 1.03 | 0.16 | 0.10 |  | -0.09 | 0.13 | 0.17 |
| 34 | 28.94 | 1.26 | 1.79 |  | -0.66 | 0.16 |  | 0.89 | 0.14 | 0.14 |  | 0.08 | 0.17 | 0.17 |
| 35 | 28.91 | 1.25 | 1.73 |  | -0.67 | 0.16 |  | 0.93 | 0.16 | 0.14 |  | 0.01 | 0.16 | 0.19 |
| 36 | 29.48 | 1.26 | 1.75 |  | -0.68 | 0.17 |  | 1.03 | 0.17 | 0.13 |  | 0.11 | 0.16 | 0.19 |
| 37 | 22.87 | 0.74 | 1.07 |  | -0.57 | 0.11 |  | 0.23 | 0.13 | 0.07 |  | -0.85 | 0.12 | 0.15 |
| 38 | 31.23 | 1.23 | 0.99 |  | -0.65 | 0.15 |  | 1.18 | 0.17 | 0.08 |  | 0.17 | 0.16 | 0.18 |
| 39 | 23.25 | 0.87 | 1.62 |  | -0.64 | 0.15 |  | 0.38 | 0.14 | 0.13 |  | -0.64 | 0.13 | 0.16 |
| 41 | 29.86 | 1.24 | 1.60 |  | -0.69 | 0.17 |  | 0.98 | 0.18 | 0.13 |  | 0.22 | 0.18 | 0.21 |
| 43 | 29.96 | 1.25 | 1.52 |  | -0.68 | 0.16 |  | 0.96 | 0.16 | 0.14 |  | 0.13 | 0.17 | 0.18 |
| 44 | 29.05 | 1.25 | 1.85 |  | -0.68 | 0.16 |  | 0.91 | 0.16 | 0.14 |  | 0.03 | 0.17 | 0.19 |
| 45 | 29.38 | 1.22 | 1.53 |  | -0.67 | 0.16 |  | 0.97 | 0.16 | 0.12 |  | 0.09 | 0.16 | 0.17 |

# **File S7. Maps of environmental and host parameters**

Spatial structures of environmental (temperature, salinity, fluorescence, turbidity and oxygen in the seawater), microbiological (total bacteria, vibrios and their concentration in the seawater and in the oyster) and biochemical parameters (energetic reserves [carbohydrate and triglyceride] and trophic markers of oysters) were described through variograms, allowing quantification of the spatial dependency and its partitioning among distance classes ^12^. Statistical models (linear, exponential and spherical) were fitted to the variograms to produce interpolated maps by kriging for each variable and for each visit using ISATIS ^13^. These maps are represented on Figures S7.1 to S7.13.

The average seawater temperature increased from 11.2°C on April 30 to 20.0°C on 25 July and decreased gradually thereafter up until reaching 17.2°C on 26 September (Fig. S7.1). The seawater temperatures were relatively homogenous within the study site (the coefficient of variation varied from as low as 1.4% on 31 August to 4.9% on 26 September). However, there was a thermal gradient from the coast to the sea. This thermal gradient was generalized throughout the study area as the water warmed up between 30 April and 4 July. For instance, at sites where high frequency temperature probes were deployed, we calculated that ΔT=0.3°C between the sites 15 and 12 and ΔT=0.5°C between the sites 39 and 38. This gradient was maintained throughout the duration of the study in the Penerf area.

Salinity varied from 29.8 to 35.4 % depending on time and site (Fig. S7.2). There was an east-west salinity gradient whose amplitude varies significantly over time. Fluorescence, which is a proxy for phytoplankton biomass in the seawater, was generally higher in the Pernerf area than in the Quiberon Bay and peaked in the middle of the study area on 20 June. This phytoplankton bloom was associated with low salinity, low turbidity and elevated concentrations of vibrios in seawater (Fig. 3). Apart from the mouth of the Pénerf River, which had the highest turbidity in the spring, the overall level of turbidity in the study area was low and typical of late spring and summer (Fig. S7.4).

Concentrations of bacteria and vibrio in seawater and oysters varied spatially and temporally (Fig. S7.6-S7-9). The bacterial populations associated with the fluorescence peak on 20 June consisted mainly of vibrio species in the seawater (Fig. S7.7). Concentrations of bacteria and vibrio species in oysters were not correlated with those recorded in seawater. On average, concentrations of bacteria and vibrio species in oysters were 8 and 67 times higher than those in seawater respectively.

Energy reserves of oysters (TAG, TAG/ST and carbohydrates) decreased from 30 April to 14 June almost everywhere (Fig S7.10 and S7.11) and increased gradually from the coast to the sea thereafter. On 20 June, the spatial pattern of energy reserves of oysters reflected that of seawater fluorescence at the same time (Fig. 3). The levels of 16:1n-7/16:0, 20:5n-3/22:6n-3 and PUFA/SFA increased between 27 May and 20 June whereas 18:2n-6+18:3n-3, branched+15:0+17:0, and 18:1n-9/18:1n-7 decreased during this this period. On 20 June, some of these indicators exhibited positive (16:1n-7/16:0) or negative (18:2n-6+18:3n-3, branched+15:0+17:0, and 18:1n-9/18:1n-7) gradients from the coast to the sea (Fig. S7.12). Spatial pattern of 16:1n-7/16:0 on 14 June mirrored energy reserves of oysters and seawater fluorescence on 20 June. Growth rates of oysters (shell length, total body mass, shell mass and flesh mass) showed the lowest values along the coast and the highest values where fluorescence peaked on 20 June (Fig. S7.13).


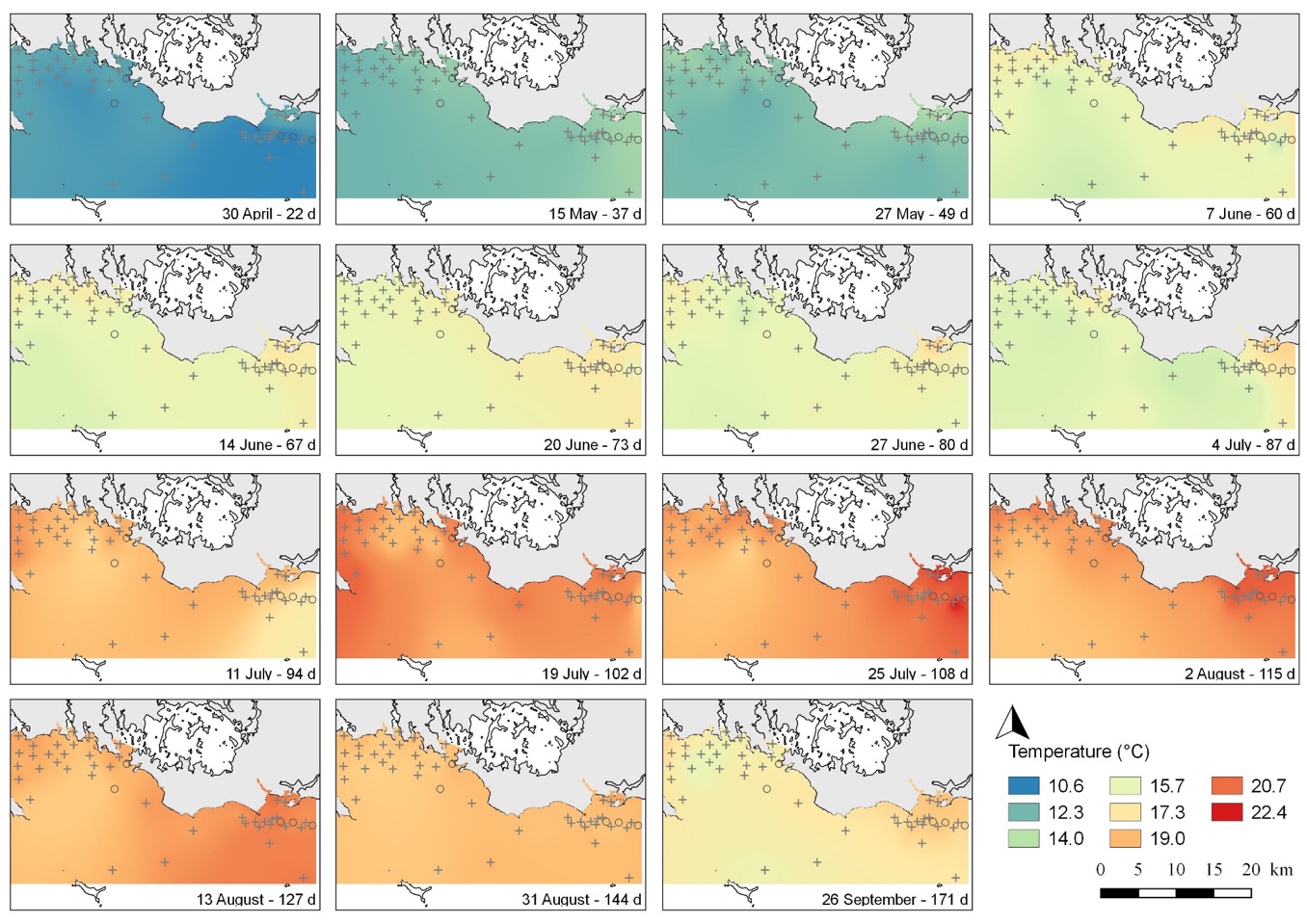
Figure S7.1 Seawater temperature in the Mor-Braz area between April and September 2013.

Figure S7.2 Seawater salinity in the Mor-Braz area between April and September 2013.
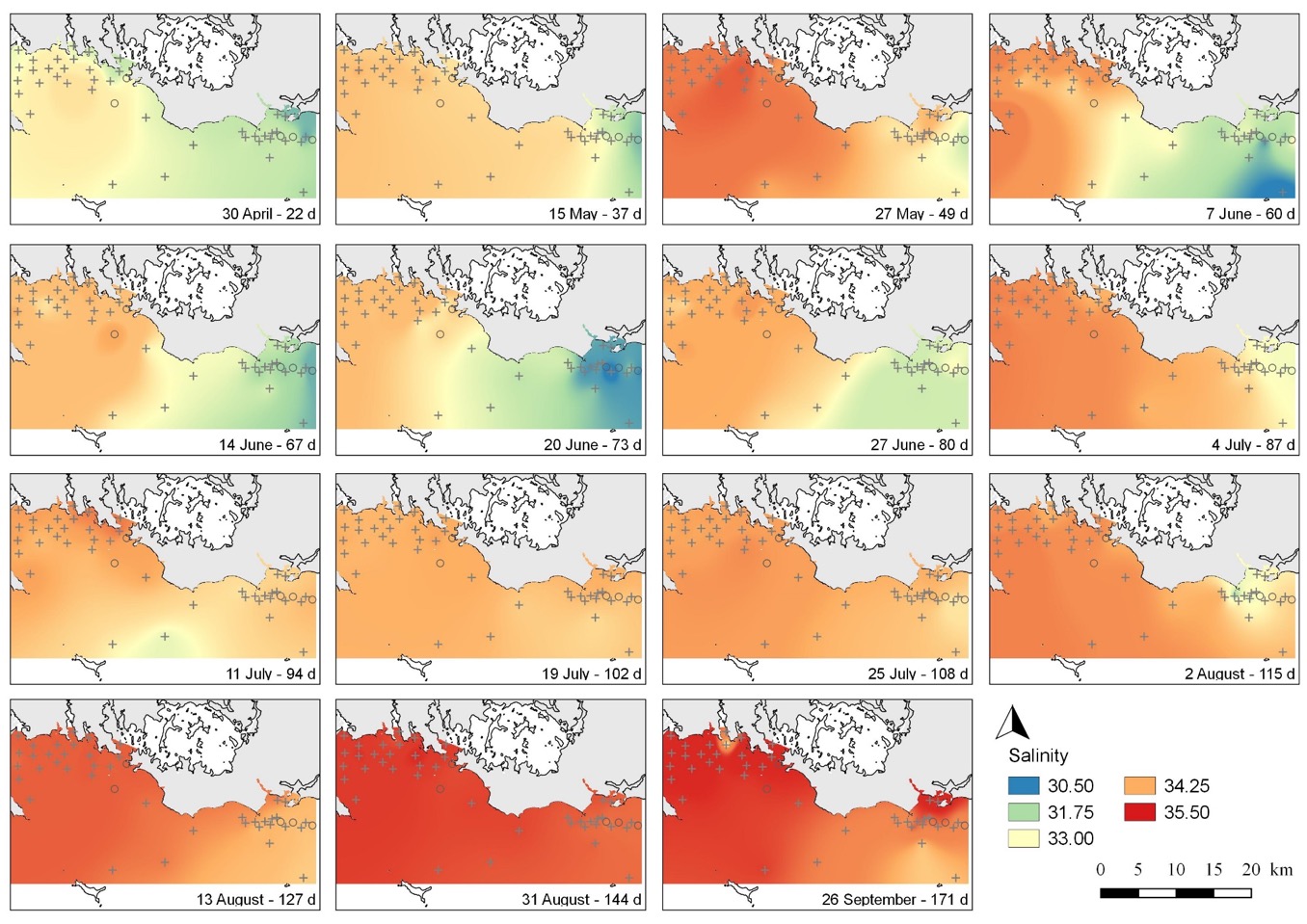


Figure S7.3 Seawater fluorescence in the Mor-Braz area between April and September 2013.
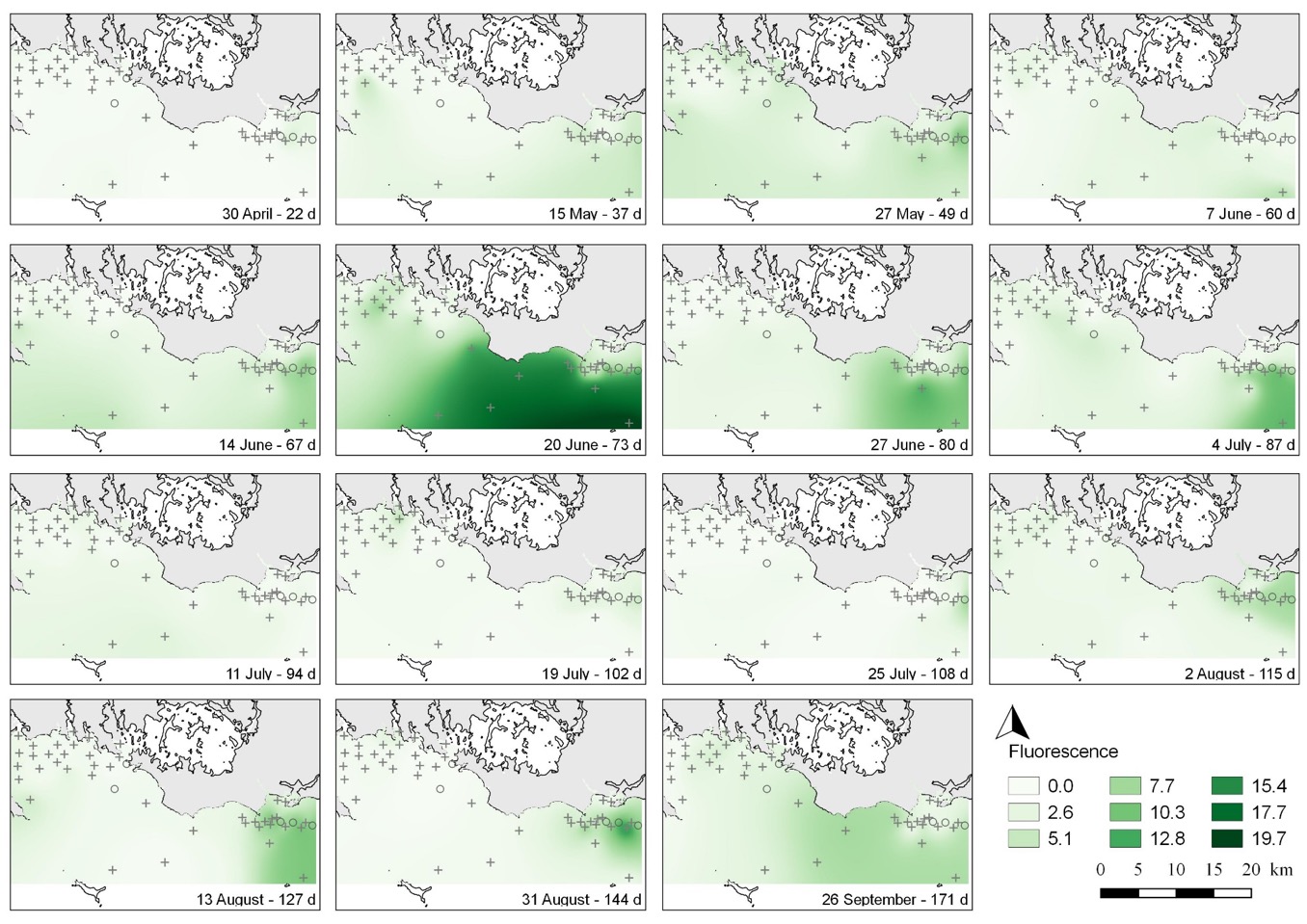


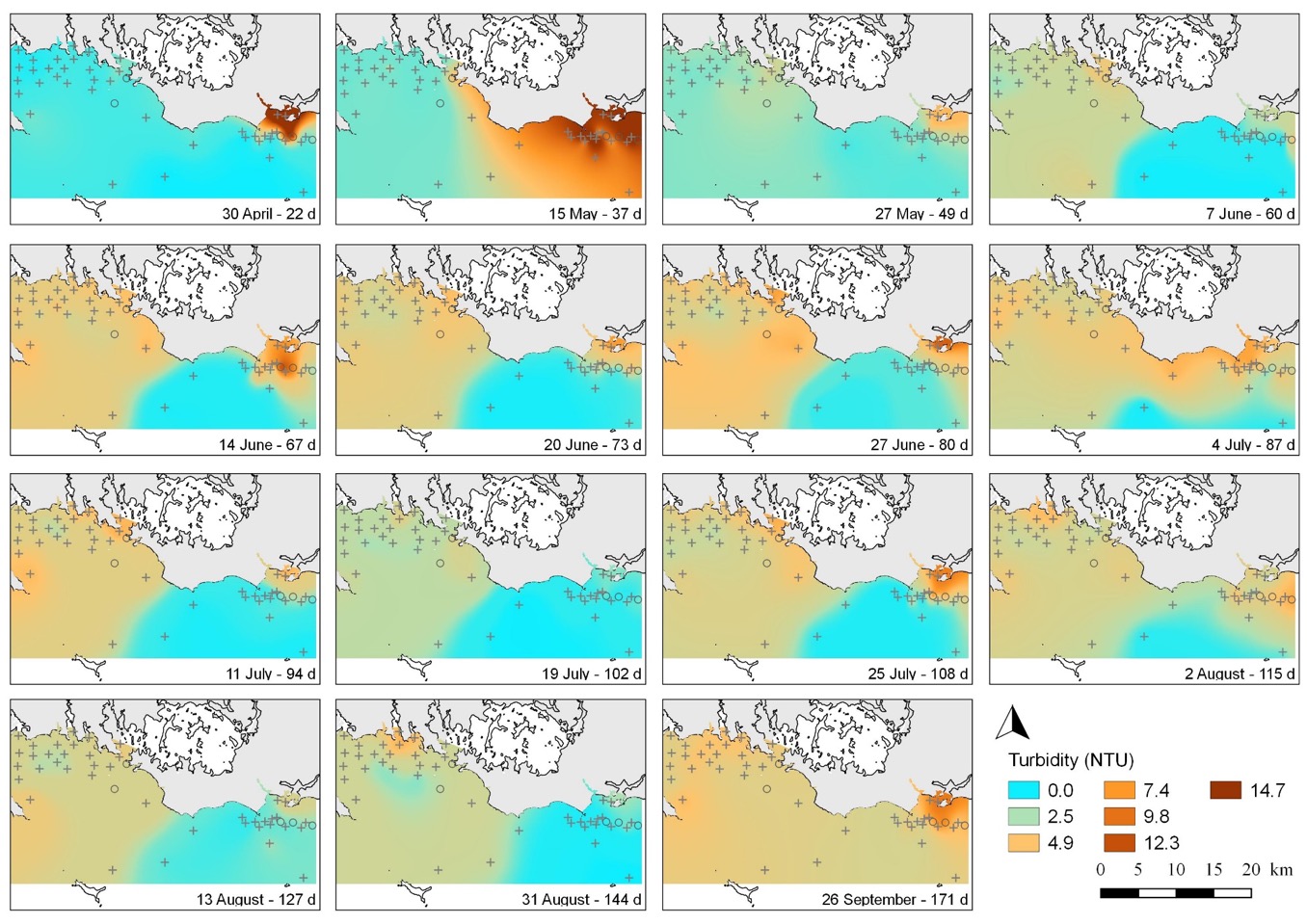
 Figure S7.4 Seawater turbidy in the Mor-Braz area between April and September 2013.


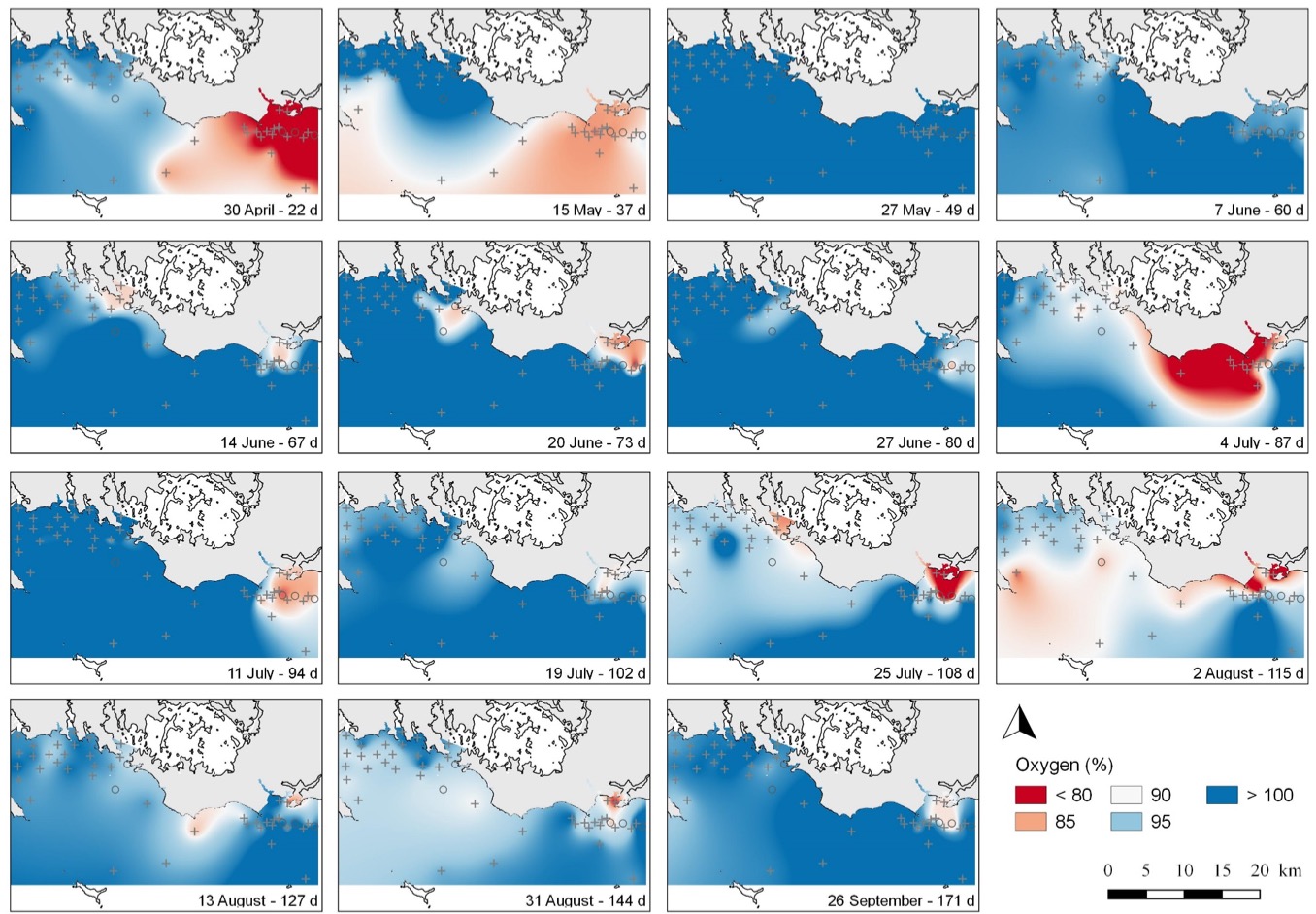
Figure S7.5 Seawater oxygen level in the Mor-Braz area between April and September 2013.

Figure S7.6 Seawater bacterial concentration in the Mor-Braz area between April and September 2013.
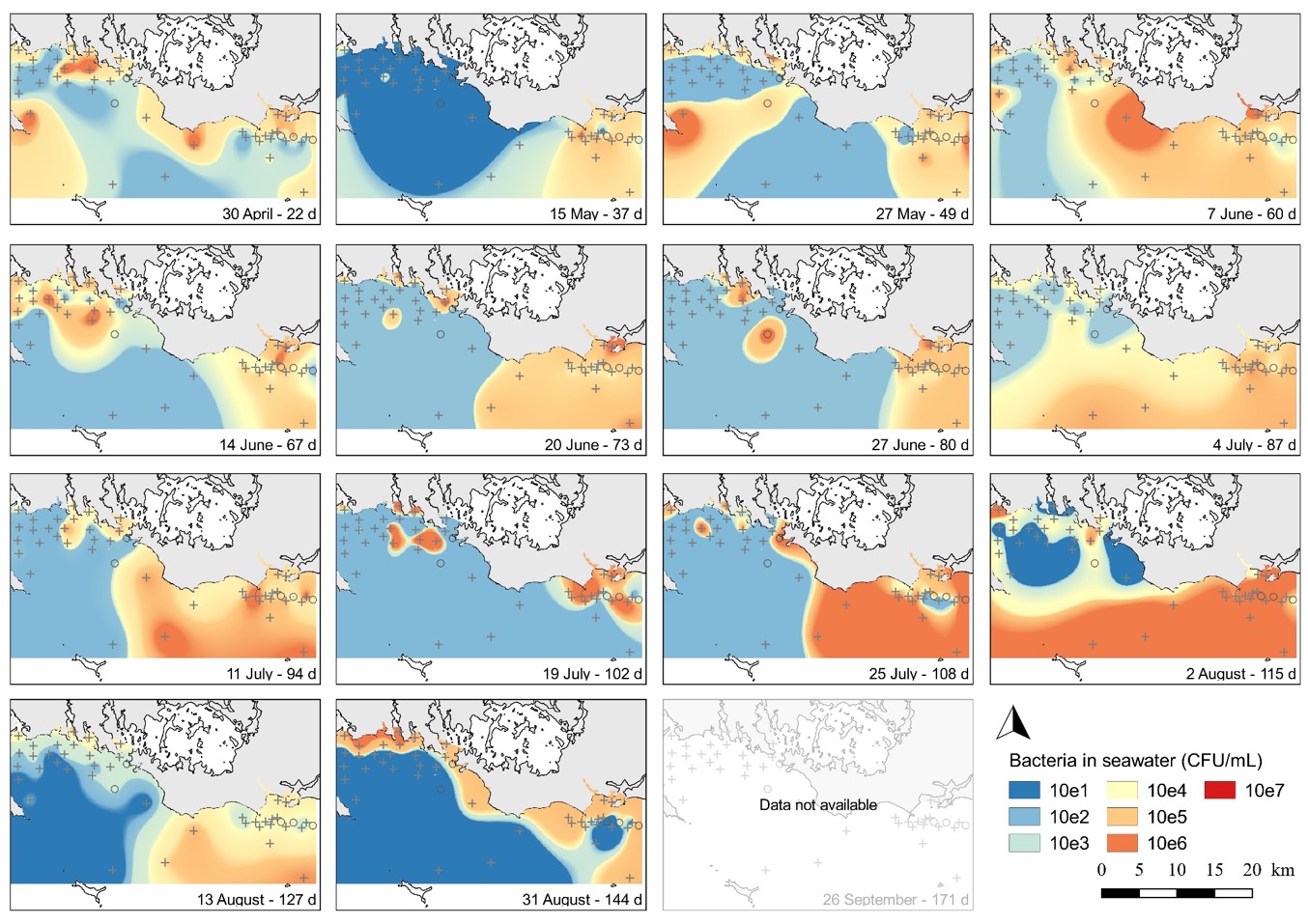


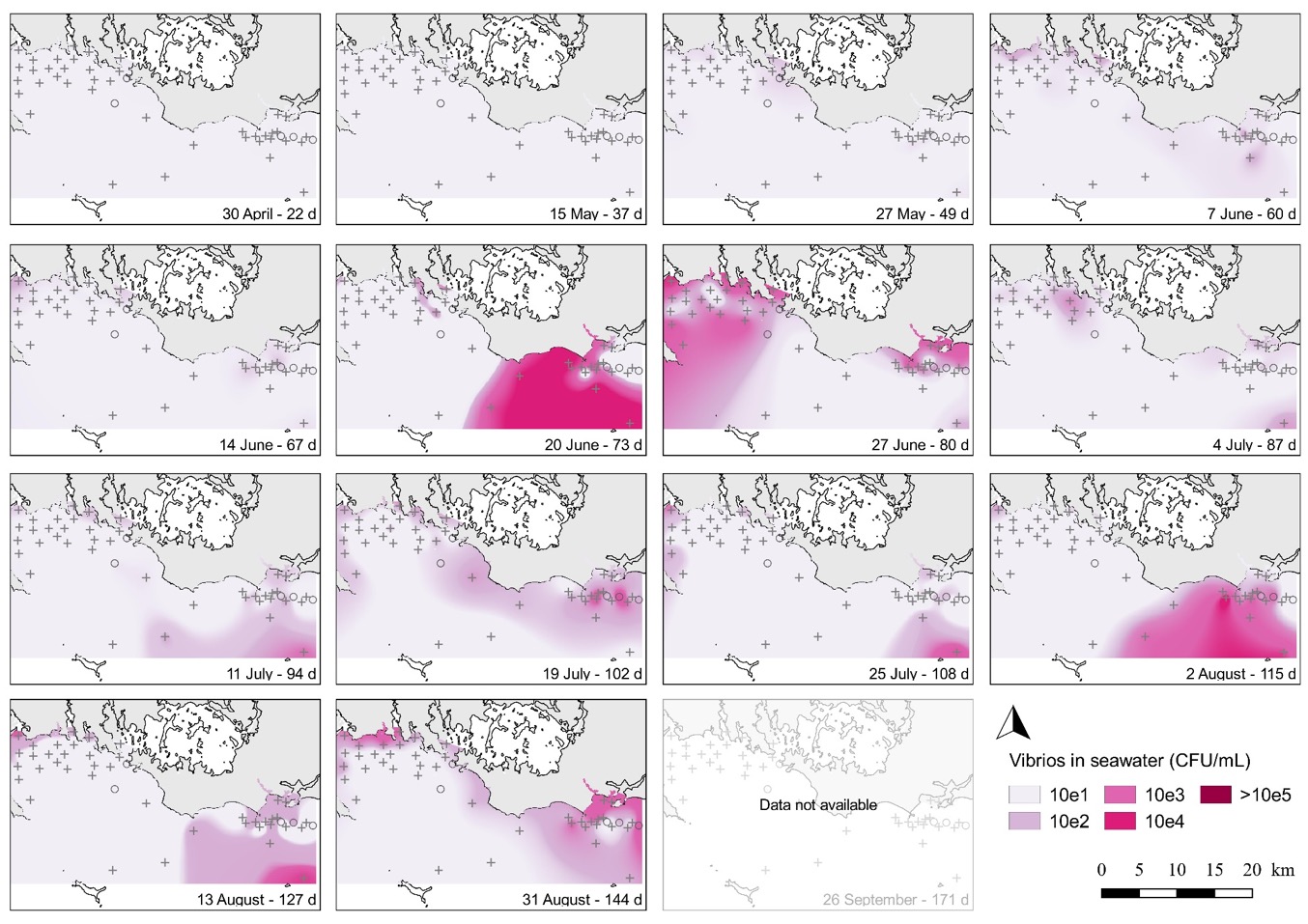
 Figure S7.7 Seawater vibrio concentration in the Mor-Braz area between April and September 2013.


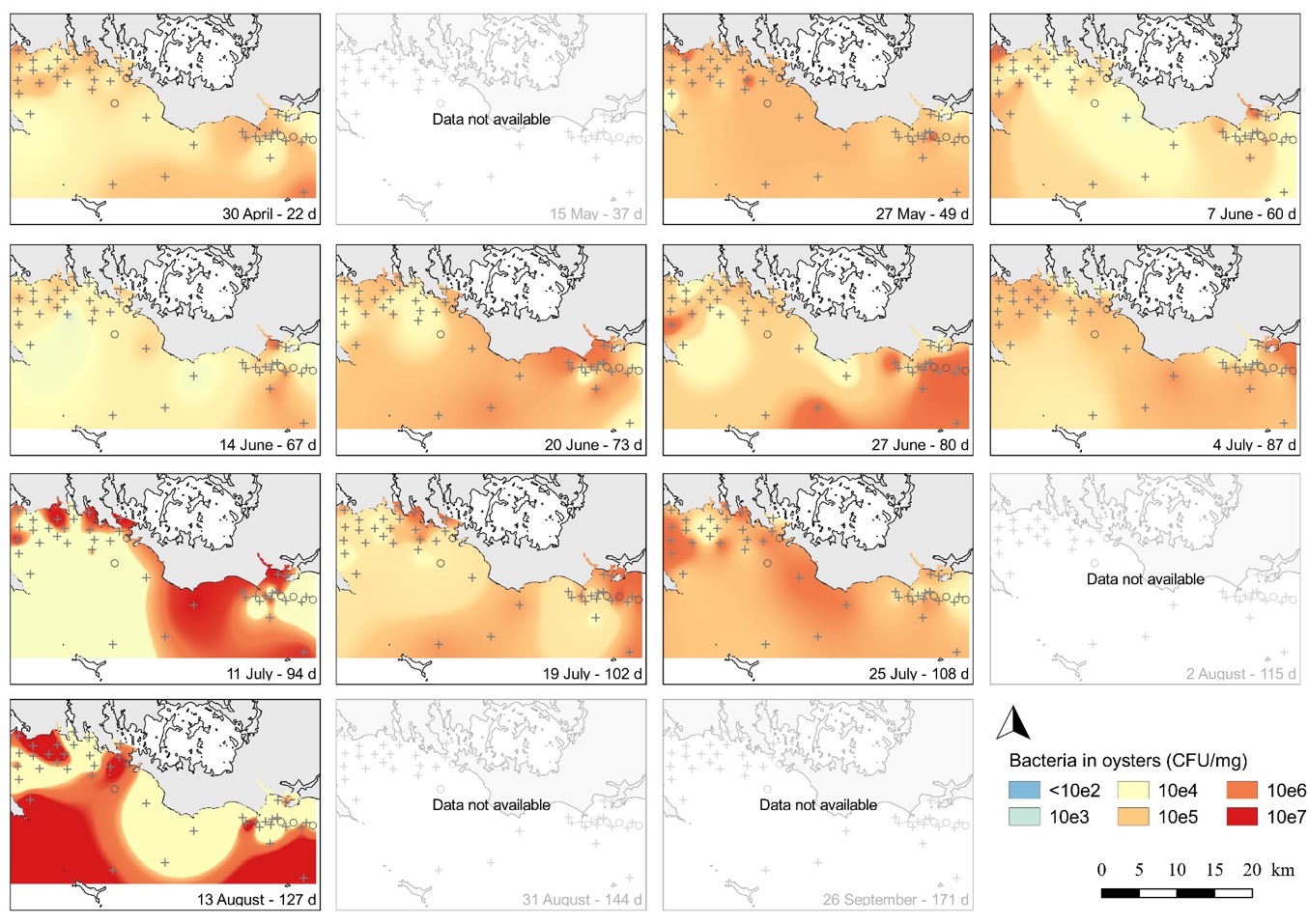
Figure S7.8 Bacterial concentration in the sentinel oysters in the Mor-Braz area between April and September 2013.


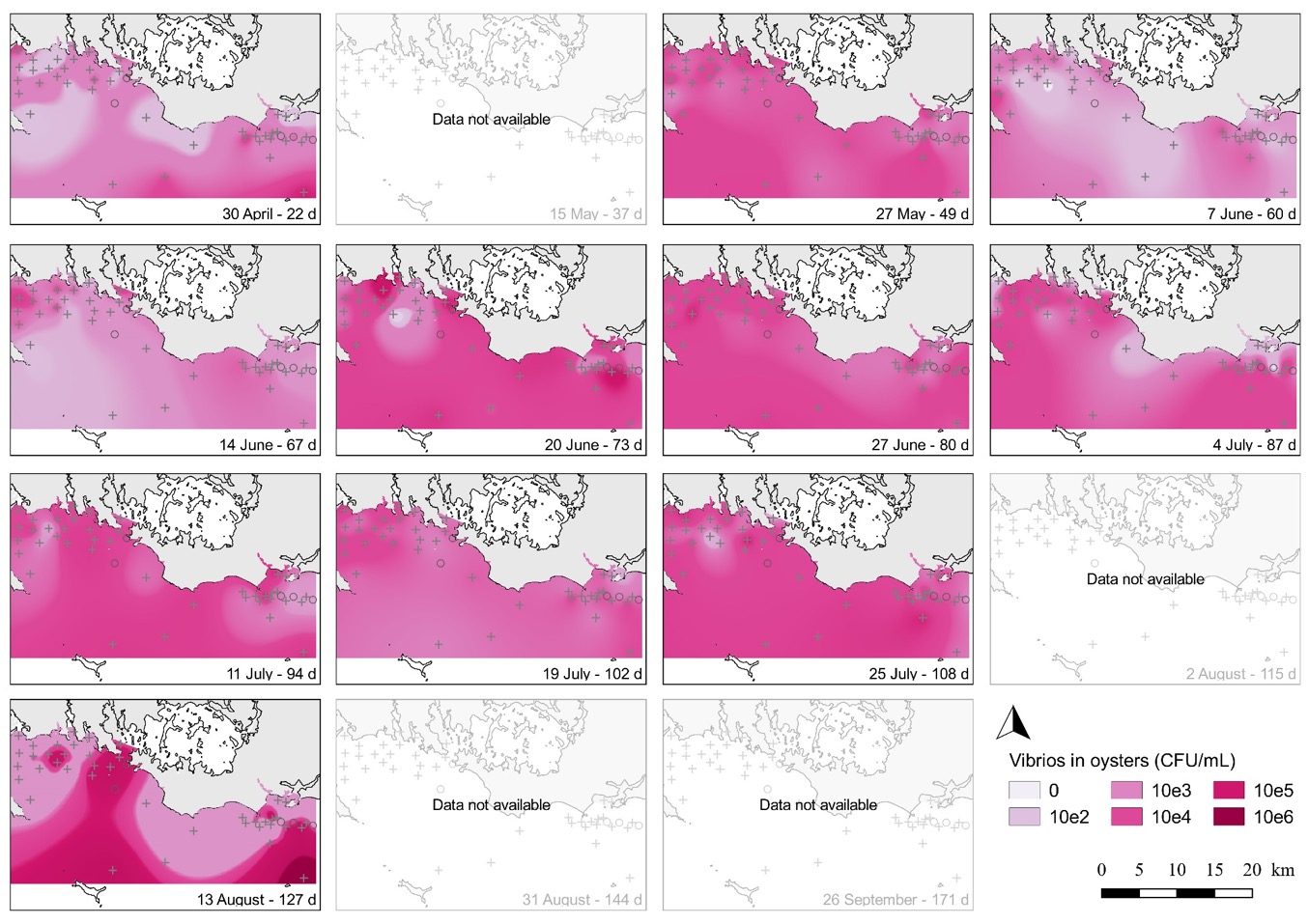
Figure S7.9 Vibrio concentration in the sentinel oysters in the Mor-Braz area between April and September 2013.


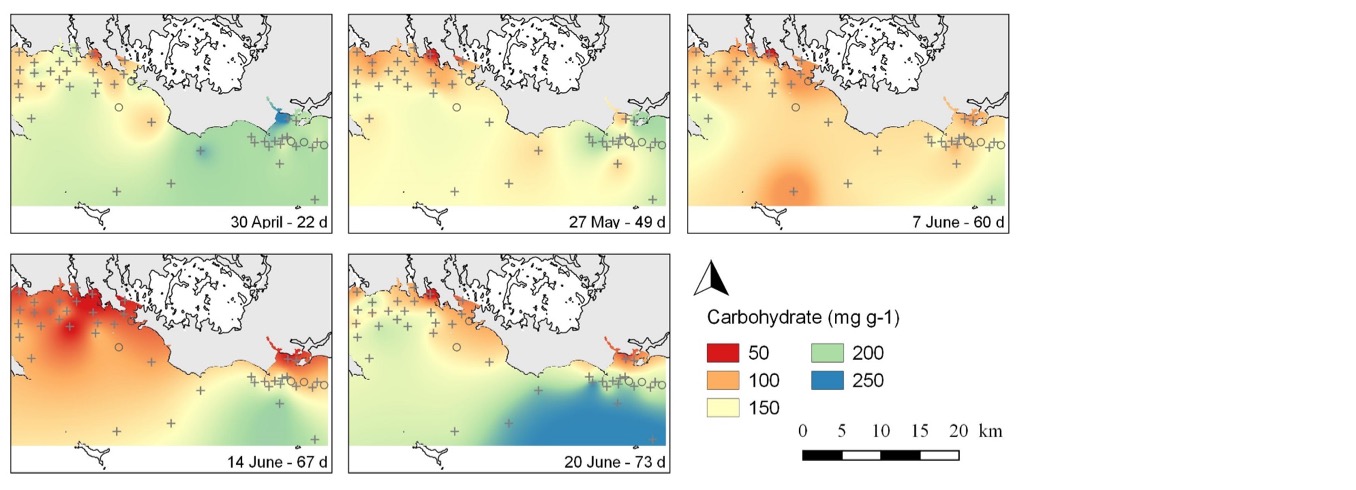
Figure S7.10 Carbohydrate concentration in the sentinel oysters in the Mor-Braz area between April and June 2013.


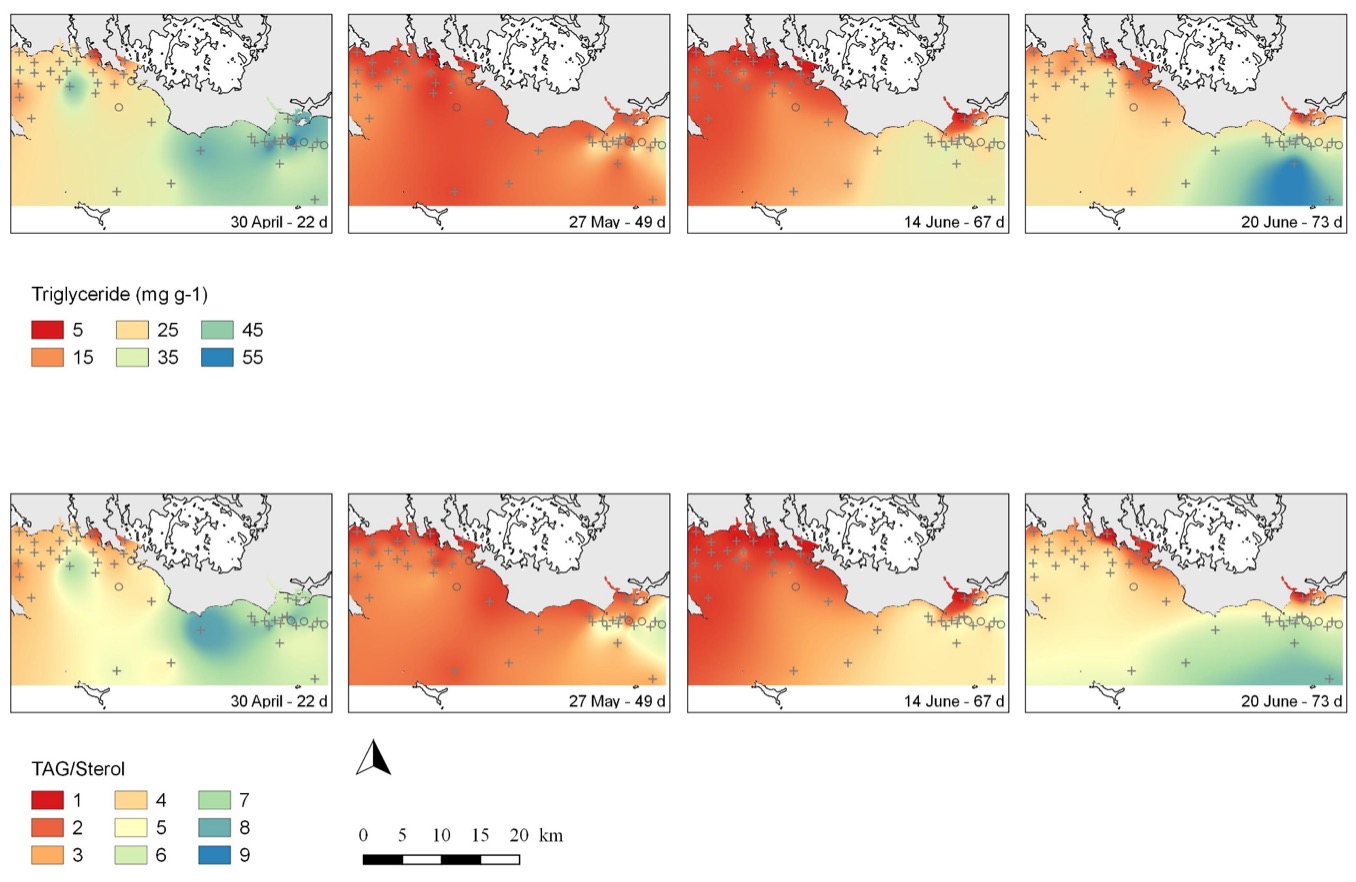
Figure S7.11 Triglyceride in the sentinel oysters in the Mor-Braz area between April and June 2013. Data were expressed as relative concentration of TAG in oyster tissues (upper panel) and as relative concentration of TAG to Sterol (lower panel)


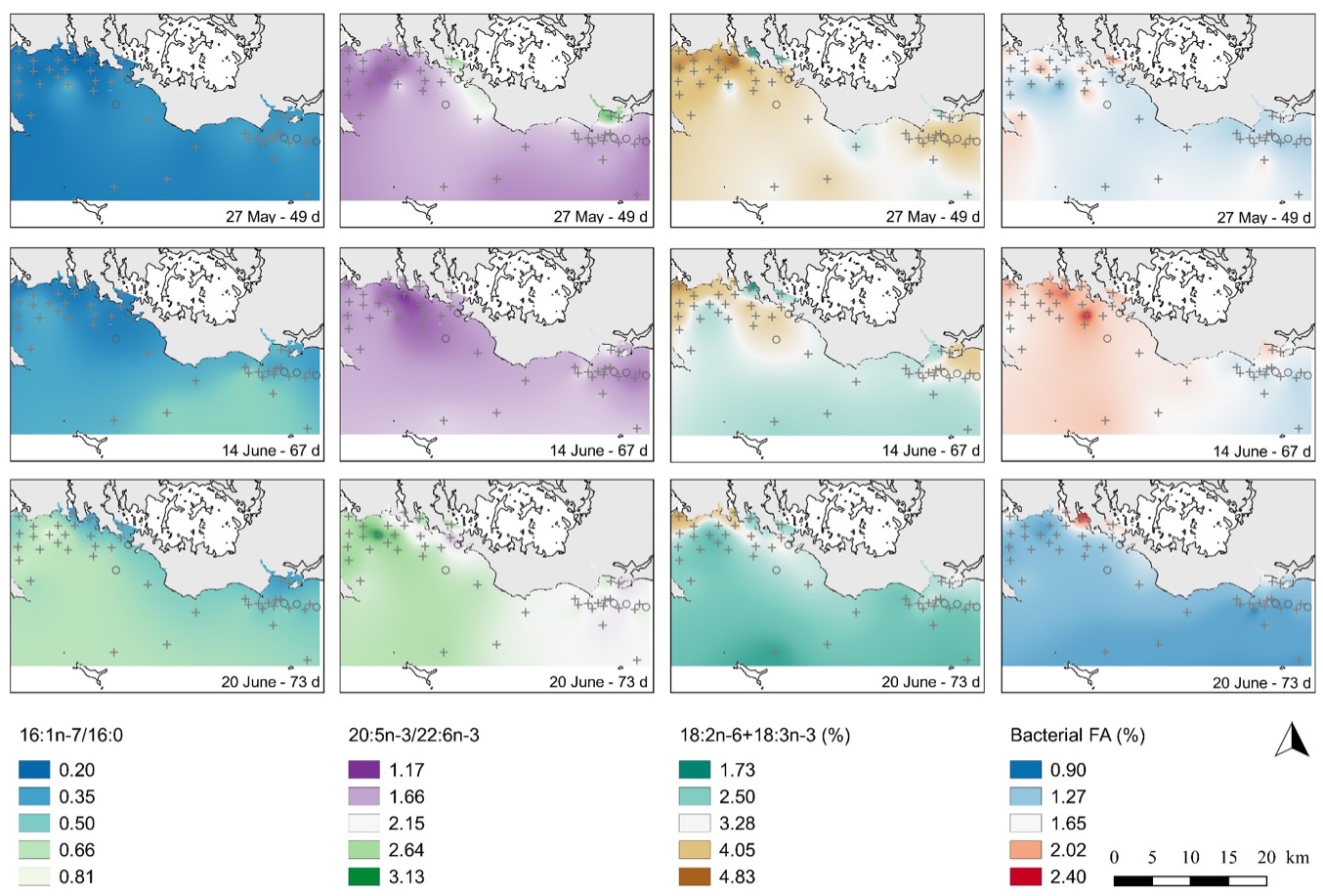
Figure S7.12 Fatty acid trophic indicators in the sentinel oysters in the Mor-Braz area in May and June 2013.

Figure S7.12
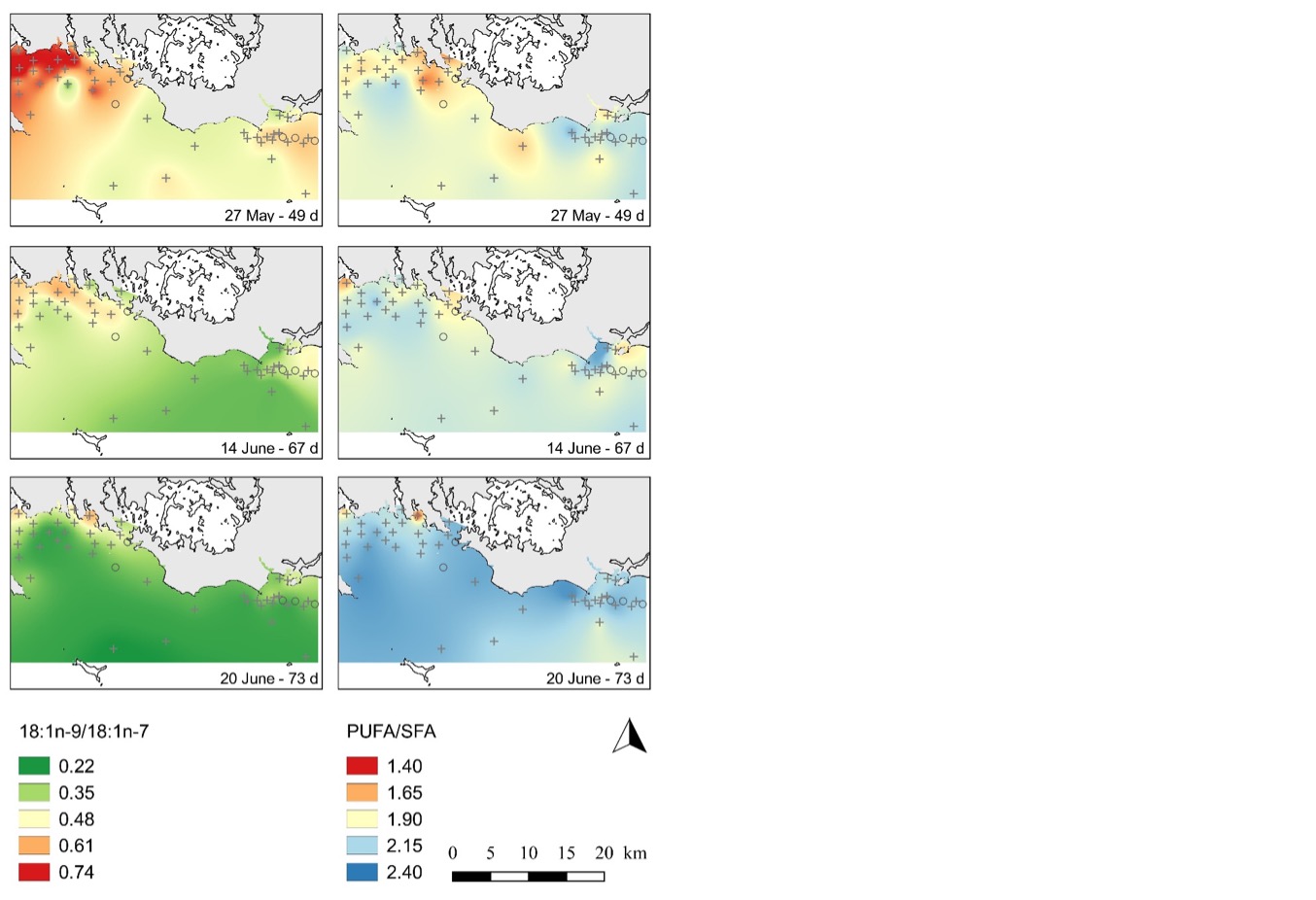
Continued.


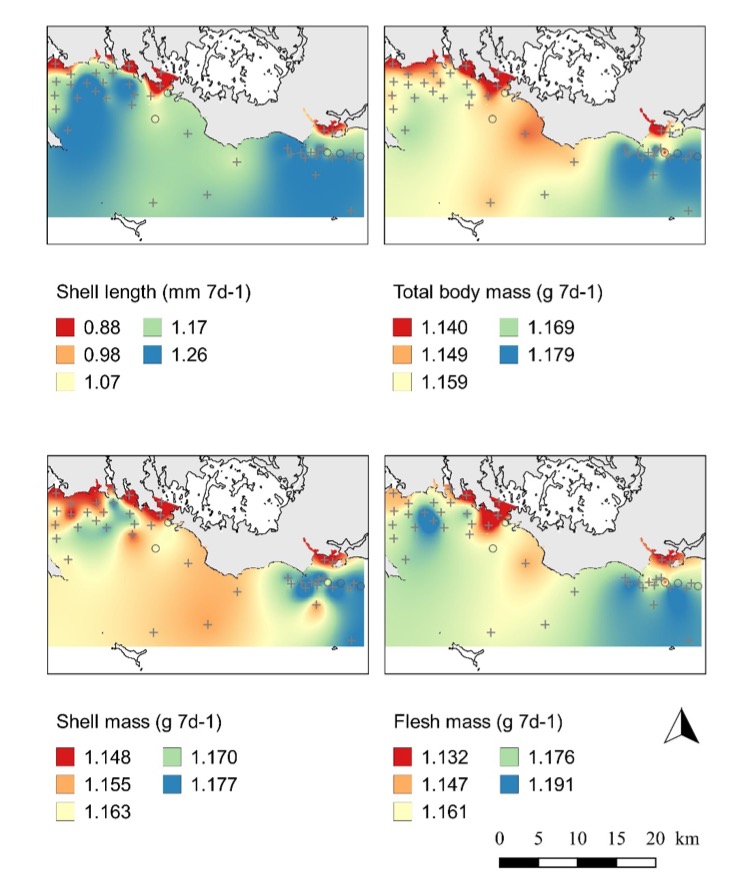
Figure S7.13 Growth rate (regression coefficients, *b_1_*) of the sentinel oysters in the Mor-Braz area in May and June 2013.

**References**

1 Petton, B., Boudry, P., Alunno-Bruscia, M. & Pernet, F. Factors influencing disease-induced mortality of Pacific oysters *Crassostrea gigas*. *Aquaculture Env. Interact.* **6**, 205-222, doi:10.3354/aei00125 (2015).

2 Pepin, J. F., Riou, A. & Renault, T. Rapid and sensitive detection of ostreid herpesvirus 1 in oyster samples by real-time PCR. *J. Virol. Methods* **149**, 269-276, doi:10.1016/j.jviromet.2008.01.022 (2008).

3 Webb, S. C., Fidler, A. & Renault, T. Primers for PCR-based detection of ostreid herpes virus-1 (OsHV-1): Application in a survey of New Zealand molluscs. *Aquaculture* **272**, 126-139, doi:10.1016/j.aquaculture.2007.07.224 (2007).

4 DuBois, M., Gilles, K. A., Hamilton, J. K., Rebers, P. A. & Smith, F. Colorimetric method for determination of sugars and related substances. *Anal. Chem.* **28**, 350-356, doi:10.1021/ac60111a017 (1956).

5 Fraser, A. J. Triacylglycerol content as a condition index for fish, bivalve and crustacean larvae. *Can. J. Fish. Aqua. Sci.* **46**, 1868-1873 (1989).

6 Metcalfe, L. D. & Schmitz, A. A. The rapid preparation of fatty acid esters for gas chromatographic analysis. *Anal. Chem.* **33**, 363-364, doi:10.1021/ac60171a016 (1961).

7 Marty, Y., Delaunay, F., Moal, J. & Samain, J.-F. Changes in the fatty acid composition of *Pecten maximus* (L.) during larval development. *J. Exp. Mar. Biol. Ecol.* **163**, 221-234, doi:10.1016/0022-0981(92)90051-B (1992).

8 Dalsgaard, J., St. John, M., Kattner, G., Muller-Navarra, D. & Hagen, W. Fatty acid trophic markers in the pelagic marine environment. *Adv. Mar. Biol.* **46**, 225-340, doi:10.1016/S0065-2881(03)46005-7 (2003).

9 Kaplan, E. L. & Meier, P. Nonparametric Estimation from Incomplete Observations. *J. Am. Stat. Assoc.* **53**, 457-481, doi:10.1080/01621459.1958.10501452 (1958).

10 Cox, D. R. Regression Models and Life Tables. *J. R. Stat. Soc. Series B Stat. Methodol.* **20**, 187-220, doi:10.1007/978-1-4612-4380-9_37 (1972).

11 Marquardt, D. W. An algorithm for least-squares estimation of nonlinear parameters. *Journal of the society for Industrial and Applied Mathematics* **11**, 431-441 (1963).

12 Legendre, P. & Legendre, L. *Numerical ecology*. Vol. 20 (Elsevier, 1998).

13 Géovariances. *ISATIS Technical References, 9th Edition, Geovariances*. 222 (2008).
